# Chronic hepatitis B virus and liver fibrosis: A mathematical model

**DOI:** 10.1371/journal.pone.0195037

**Published:** 2018-04-10

**Authors:** Avner Friedman, Nourridine Siewe

**Affiliations:** 1 Mathematical Biosciences Institute & Department of Mathematics, The Ohio State University, Columbus, Ohio, United States of America; 2 National Institute for Mathematical and Biological Synthesis, University of Tennessee, Knoxville, Tennessee, United States of America; Ospedale San Raffaele, ITALY

## Abstract

Hepatitis B virus (HBV) infection is a liver disorder that can result in cirrhosis, liver failure and hepatocellular carcinoma. HBV infection remains a major global health problem, as it affects more 350 million people chronically and kills roughly 600,000 people annually. Drugs currently used against HBV include IFN-*α* that decreases viremia, inflammation and the growth of liver fibrosis, and adefovir that decreases the viral load. Each of these drugs can have severe side-effects. In the present paper, we consider the treatment of chronic HBV by a combination of IFN-*α* and adefovir, and raise the following question: What should be the optimal ratio between IFN-*α* and adefovir in order to achieve the best ‘efficacy’ under constraints on the total amount of the drugs; here the efficacy is measured by the reduction of the levels of inflammation and of fibrosis? We develop a mathematical model of HBV pathogenesis by a system of partial differential equations (PDEs) and use the model to simulate a ‘synergy map’ which addresses the above question.

## Introduction

Hepatitis B virus (HBV) infection is a liver disorder that can result in cirrhosis, liver failure, and hepatocellular carcinoma; it is one of the most prevalent infectious diseases associated with human liver diseases, including acute, fulminant and chronic hepatitis [1]. Despite the availability of HBV vaccine and the development of antiviral therapies, HBV infection remains a major global health problem. Chronic HBV affects more than 350 million people of whom roughly 600,000 die annually from HBV-related liver diseases [1–3].

To understand the virus biology and pathogenesis in HBV-infected patients, several animal models have been developed to mimic hepatic HBV infection and the immune response against HBV [1, 4, 5]. However, as observed in [1], the narrow host range of HBV infection and lack of a full immune response spectrum in animal models remain significant limitations. For this reason, it may be useful to develop a mathematical model of the progression of HBV, which includes the host response to treatment. In the present paper we introduce such a model and use it to study the effect of a combination therapy with two currently used drugs, interferon alpha (IFN-*α*) [5–7] and adefovir [8–11]. The model shows that it may be possible to reduce HBV infection and viral load while, at the same time, decrease inflammation and control liver fibrosis.

Monotherapy of HBV infection with IFN-*α* decreases viremia, inflammation and the progression of liver fibrosis. However, only a small fraction of patients respond positively to treatment [5, 6]. Monotherapy with adefovir decreases the viral load by blocking reverse transcriptase, an enzyme that is crucial for the reproduction of HBV in the body [8, 12, 13].

Both IFN-*α* and adefovir can have severe side-effects when administered alone, including brain disorder that may lead to a stroke, a heart attack or lung fibrosis for IFN-*α* [14], and kidney damage, a low amount of phosphate in the blood, a stroke or pancreatitis for adefovir [15]. A combination therapy with smaller dose may be as effective, or even more effective, than monotherapy with a larger dose if there is a ‘positive synergy’ between the two drugs. We shall use our mathematical model to determine the synergy between IFN-*α* and adefovir.

In their study of HBV infection in a humanized mouse model where the mice are inoculated with clinical isolates HBV *in vivo*, the authors in [16] showed that a persistence of liver disease due to HBV infection is associated with infiltration of M2 macrophages. They found that chronic HBV infection in the liver of humanized mice is associated with human hepatic stellate cells (HSCs) activation/infection and human liver fibrosis. It was also observed in [16] that infection of M1 and M2 macrophages with HBV results in increasing level of IL-10 and decreasing level of IL-12. Although the authors in [16] do not demonstrate that HBV infects macrophages, they show that inoculation of macrophage cultures with HBV results in macrophage activation, however the mechanism of activation is unknown. Since the possibility that HBV could infect macrophages remains to be conclusively proven, we do not assume that macrophage infection is a major source of virus as compared to HSCs.

## Methods

In the course of chronic HBV infection, M1 macrophages produce pro-inflammatory cytokines IL-6 [17–19], IL-12 [17, 20, 21] and TNF-*α* [22, 23], while M2 macrophages produce anti-inflammatory cytokines IL-1*β* [17, 18, 20, 24], IL-4 [25, 26], IL-10 [17–19, 27, 28], IL-13 [17–19, 27–29] and TGF-*β* [30], and chemokine PDGF [31, 32]. The HBV environment includes T cells: Th1 and Th2. Th1 is activated by IL-12 [33] and it produces IL-2 [17], IFN-*α* [34] and IFN-*γ* [17, 20, 35]. Th2 is activated by IL-4 [36]. Th2 is activated by IL-4 [37] and it produces IFN-*α* [38], IL-4 [25, 26], IL-10 [27, 28] and IL-13 [27, 28, 39]. Th1 and Th2 are mutually antagonistic [36, 40, 41]. In addition to macrophages and T cells, the mathematical model will include other types of cells closely associated with chronic HBV: hepatic stellate cells (HSCs), fibroblasts and myofibroblasts. HSCs enhance the proliferation of fibroblasts by producing hyaluronic acid [42–44]. Fibroblasts are transformed into myofibroblasts by PGDF and TGF-*β* [30, 45–47]. Fibroblast and, more effectively, myofibloblast, produce collagen [47–50]. MMP produced by M2 macrophages disrupts collagen cross-linking and increases scarring, especially under excessive collagen concentrations [31, 32, 51]. Since M2 macrophages produce TGF-*β* and PDGF which enhance the presence of fibroblasts, M2 macrophages contribute to the progression of fibrosis. In chronic HBV, the virus infects both M1 and M2 macrophages, as well as hepatic stellate cells. The infected cells secrete C-C Motif Chemokine Ligand 3 (CCL3) [45, 52, 53] which attracts macrophages into the liver [31, 54], resulting in increased inflammation. The replication of the intracellular virus is blocked by several cytokines, including IFN-*α*, TNF-*α*, TGF-*β* and IFN-*γ* [20, 35, 55].

**Fig 1:**
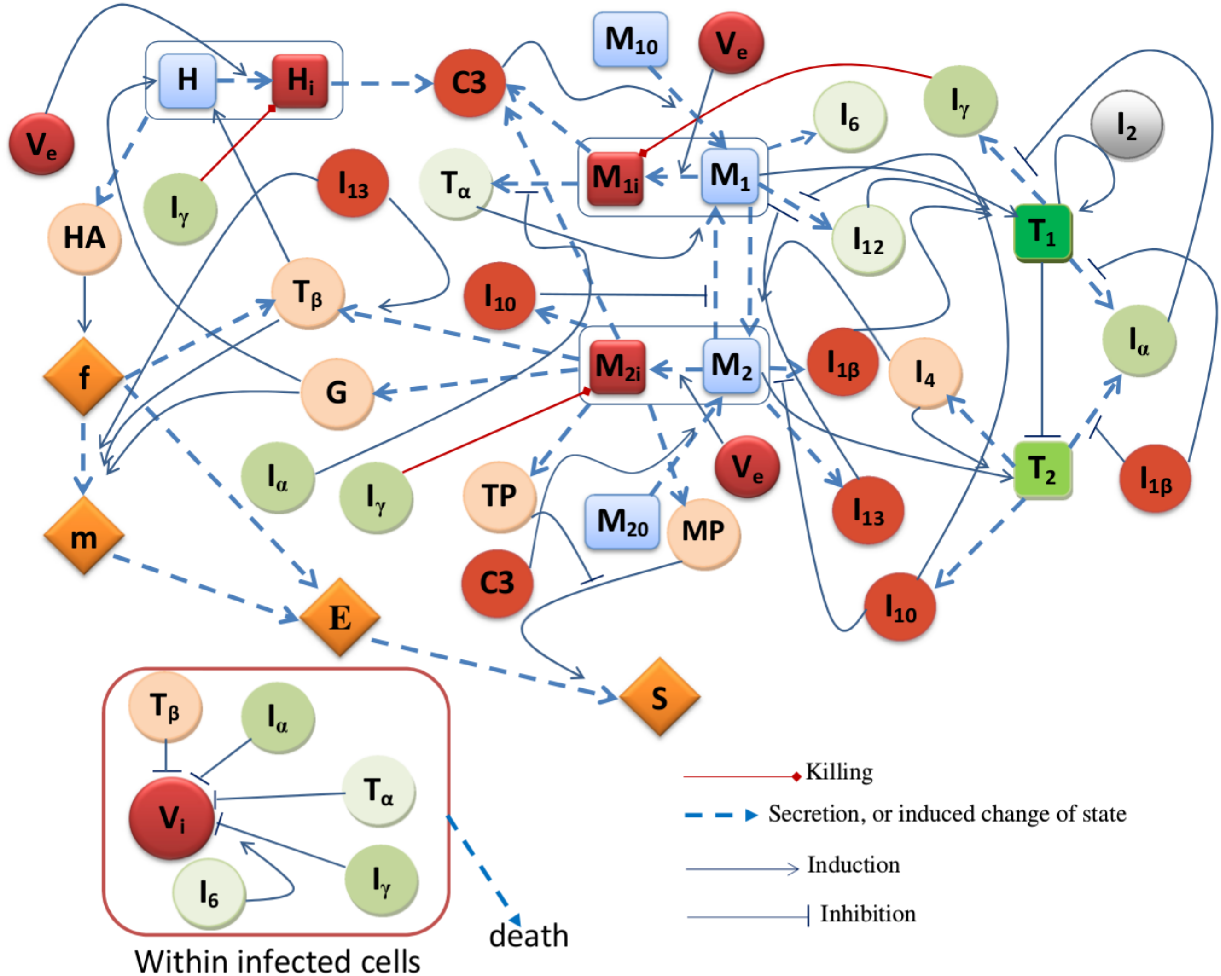
Diagram of interactions between cells, virus and cytokines in hepatitis B.

A schematic diagram of our model is shown in Fig 1, where the nodes are defined in Table 1. We represent the cells by square nodes, but the fibrosis formation pathway by diamond nodes, and the virus and cytokines by circular nodes. The nodes with reddish colors (red and orange) indicate the variables that include and/or induce infection or fibrosis formation. the nodes with greenish colors (blue, green and silver) indicate the variables that counteract the infection. We shall use our model to compare the efficacy of treatment by a single agent (IFN-*α* or adefovir) with a combination, whereby the amount of each agent is less than in the monotherapy. We shall accordingly develop a ‘synergy map’ which addresses the question of achieving a desired target of efficacy in a combination therapy while using the smallest dose of these two drugs.

**Table 1:** Variables of the model. All densities and concentrations are in units of g/cm^3^.

Variables	Descriptions	Variables	Descriptions
*H*	density of healthy HSCs	*H*_*i*_	density of infected HSCs
*M*_1_	density of healthy M1 macrophages	*M*_1*i*_	density of infected M1 macrophages
*M*_2_	density of healthy M2 macrophages	*M*_2*i*_	density of infected M2 macrophages
*T*_1_	Th1 cell density	*T*_2_	Th2 cell density
*f*	density of fibroblast	*m*	density of myofibroblast
*ρ*	density of ECM	*S*	scar density
*V*_*e*_	density of external virus	*V*_*i*2_	density of virus in *M*_2*i*_
*V*_*i*1_	density of virus in *M*_1*i*_	*V*_*iH*_	density of virus in *H*_*i*_
*G*	concentration of PDGF	*H*_*A*_	concentration of hyaluronic acid
*I*_1*β*_	concentration of IL-1*β*	*I*_2_	concentration of IL-2
*I*_4_	concentration of IL-4	*I*_6_	concentration of IL-6
*I*_10_	concentration of IL-10	*I*_12_	concentration of IL-12
*I*_13_	concentration of IL-13	*I*_*α*_	concentration of IFN-*α*/*β*
*I*_*γ*_	concentration of IFN-*γ*	*T*_*α*_	concentration of TNF-*α*
*T*_*β*_	concentration of TGF-*β*	*M*_*P*_	concentration of MMP
*T*_*P*_	concentration of TIMP	*C*_3_	concentration of CCL3

Mathematical models for population dynamics of HBV transmission have been developed [56–63]. In [62], the authors used a SEIR-type mathematical model by a system of ordinary differential equations (ODEs) to study the dynamics of HBV infection under administration of vaccination and treatment. A compartmental mathematical model by a system of partial differential equations (PDEs) to predict the dynamics of HBV transmission and evaluate the long-term effectiveness of the vaccination program was developed in [63]. Another class of models that consider uninfected hepatocytes, infected hepatocytes and free virus have been developed to analyze changes in HBV levels during drug therapy [56–61]. Techniques in [58, 59] where used in [56, 60, 61] to develop mathematical models by a system of ordinary differential equations to understand the factors that govern HBV infection dynamics, and to analyze the immune mechanisms responsible for viral clearance in the case of acute HBV infection. They found that the viral load initially increases exponentially, then decreases and seems to approach a plateau after some time. Interestingly, they obtained that the number of infected cells, the number of uninfected cells and the number of CD8^+^ T cells increase in acute HBV infection, leading to clearance of the viral load. A more recent model by Friedman and Hao [64] uses reaction-diffusion equations to study the evolution of fibrosis in the liver, without specifically targeting the cause of the disease.

## Mathematical model

In this section, we develop a mathematical model of liver fibrosis due to infection by HBV. In our model that builds on the model in [64], infection takes place in some region *Ω* of the liver. The variables used in the model are given in Table 1. These variables satisfy a system of PDEs in *Ω*.

**Equation for macrophage density.** The equation for the density of healthy M1 macrophages, not yet including a source, is given by

$$\begin{array}{ccc} \frac{\partial M_{1}}{\partial t}-D_{M}\Delta M_{1} & = & -\underset{\text{chemotaxis}}{\underset{︸}{\nabla \cdot (M_{1}\chi_{C_{3}}\nabla C_{3})}}+\underset{M_{2}\to M_{1}}{\underset{︸}{\lambda_{M_{2}M_{1}}\frac{\varepsilon_{1}}{\varepsilon_{1}+\varepsilon_{2}}M_{2}}}-\underset{M_{1}\to M_{2}}{\underset{︸}{\lambda_{M_{1}M_{2}}\frac{\varepsilon_{2}}{\varepsilon_{1}+\varepsilon_{2}}M_{1}}}\\ & & -\underset{M_{1}\to M_{1i}}{\underset{︸}{\lambda_{M_{1}M_{1i}}V_{e}M_{1}}}-\underset{\text{death}}{\underset{︸}{\mu_{M_{1}}M_{1}}}, \end{array}$$

where

$$\begin{array}{ccc} \varepsilon_{1} & = & \left(\lambda_{MI_{\gamma }}\frac{I_{\gamma }}{I_{\gamma }+K_{\gamma }}+\lambda_{MT_{\alpha }}\frac{T_{\alpha }}{T_{\alpha }+K_{T_{\alpha }}}\right)\frac{1}{1+I_{10}/{\tilde{K}}_{10}},\\ \varepsilon_{2} & = & \lambda_{MI_{4}}\frac{I_{4}}{I_{4}+K_{4}}+\lambda_{MI_{13}}\frac{I_{13}}{K_{13}+I_{13}}. \end{array}$$

The term ∇ ⋅ (*M*_1_ *χ*_*C*_3__∇*C*_3_) is the chemotactic effect of *C*_3_ on M1 macrophages; *χ*_*C*_3__ is the chemotactic coefficient. M2 macrophages can become M1 macrophages under the influence of IFN-*γ* and TNF-*α*, a process resisted by IL-10 [65–67], and M1 macrophages can become *M*_2_ macrophages under the influence of IL-4 and IL-13 [29, 68]. The fourth term on the right-hand side is the infection of M1 macrophages by external virus, *V*_*e*_.

Infected cells produced chemokine CCL3 [45, 52, 53] in order to attract immune response. Monocytes circulate in the blood. They are attracted to the liver tissue by CCL3 [31, 54], and then they differentiate into either M1 or M2 macrophages [69–71]. We denote *M*_10_ and *M*_20_ the densities of the monocytes from the vascular system which differentiate into M1 and M2 macrophages, respectively. Accordingly, on the boundary of each blood capillary, there is an influx of M1 macrophages into the tissue,

$$\begin{array}{c} D_{M}\frac{\partial M_{1}}{\partial n}+\tilde{\beta }\left(C_{3}\right)\left(M_{10}-M_{1}\right)=0, \end{array}$$

where $\tilde{\beta }\left(C_{3}\right)$ is an increasing function of *C*_3_. Using a homogenization method as in [52], we can replace the boundary conditions on the blood capillaries by a source term in the tissue, which we take to be $\beta \left(C_{3}\right)=\beta \frac{C_{3}}{K_{C_{3}}+C_{3}}$, where *K*_*C*_3__ is a constant. Hence the final equation for the density of M1 macrophages is the following:

$$\begin{array}{ccc} \frac{\partial M_{1}}{\partial t}-D_{M}\Delta M_{1} & = & \beta \left(C_{3}\right)\left(M_{10}-M_{1}\right)-\underset{\text{chemotaxis}}{\underset{︸}{\nabla \cdot (M_{1}\chi_{C_{3}}\nabla C_{3})}}+\underset{M_{2}\to M_{1}}{\underset{︸}{\lambda_{M_{2}M_{1}}\frac{\varepsilon_{1}}{\varepsilon_{1}+\varepsilon_{2}}M_{2}}}\\ & & -\underset{M_{1}\to M_{2}}{\underset{︸}{\lambda_{M_{1}M_{2}}\frac{\varepsilon_{2}}{\varepsilon_{1}+\varepsilon_{2}}M_{1}}}-\underset{M_{1}\to M_{1i}}{\underset{︸}{\lambda_{M_{1}M_{1i}}V_{e}M_{1}}}-\underset{\text{death}}{\underset{︸}{\mu_{M_{1}}M_{1}}}. \end{array}$$ (1)

The density *M*_1*i*_ of infected M1 macrophage satisfies the following equation:

$$\begin{array}{ccc} \frac{\partial M_{1i}}{\partial t}-D_{M}\Delta M_{1i} & = & \underset{M_{1}\to M_{1i}}{\underset{︸}{\lambda_{M_{1}M_{1i}}V_{e}M_{1}}}-\underset{\text{death}}{\underset{︸}{\mu_{M_{1}}\left(1+\mu_{V_{i1}}V_{i1}\right)M_{1i}}}, \end{array}$$ (2)

where we assume that the death rate of *M*_1*i*_ is increased due to the viral load represented by *μ*_*V*_*i*1__ *V*_*i*1_.

The density of healthy M2 macrophages satisfies an equation similar to Eq (1):

$$\begin{array}{ccc} \frac{\partial M_{2}}{\partial t}-D_{M}\Delta M_{2} & = & \underset{\text{source}}{\underset{︸}{\beta \left(C_{3}\right)\left(M_{20}-M_{2}\right)}}-\underset{\text{chemotaxis}}{\underset{︸}{\nabla \cdot (M_{2}\chi_{C_{3}}\nabla C_{3})}}+\underset{M_{1}\to M_{2}}{\underset{︸}{\lambda_{M_{1}M_{2}}\frac{\varepsilon_{2}}{\varepsilon_{1}+\varepsilon_{2}}M_{1}}}\\ & & -\underset{M_{2}\to M_{1}}{\underset{︸}{\lambda_{M_{2}M_{1}}\frac{\varepsilon_{1}}{\varepsilon_{1}+\varepsilon_{2}}M_{2}}}-\underset{M_{2}\to M_{2i}}{\underset{︸}{\lambda_{M_{2}M_{2i}}V_{e}M_{2}}}-\underset{\text{death}}{\underset{︸}{\mu_{M_{2}}M_{2}}} \end{array}$$ (3)

The third, fourth and fifth terms in the right-hand side of Eq (3) are complementary to the corresponding terms in Eq (1).

The density *M*_2*i*_ of infected M2 macrophages satisfies an equation similar to Eq (2):

$$\begin{array}{ccc} \frac{\partial M_{2i}}{\partial t}-D_{M}\Delta M_{2i} & = & \underset{M_{2}\to M_{2i}}{\underset{︸}{\lambda_{M_{2}M_{2i}}V_{e}M_{2}}}-\underset{\text{death}}{\underset{︸}{\mu_{M_{2}}\left(1+\mu_{V_{i2}}V_{i2}\right)M_{2i}}}, \end{array}$$ (4)

where we assume that the death rate of *M*_2*i*_ is increased due to the viral load *μ*_*V*_*i*2__ *V*_*i*2_.

**Equations for hepatic stellate cells (HSCs) density.** In homeostasis, HSCs have a source *A*_*H*_ and death rate *μ*_*H*_. In HBV, the proliferation of the healthy population of HSCs is enhanced by PDGF and TGF-*β* [45, 53, 72] and is reduced when they become infected by the extracellular virus. Hence the equation for *H* is the following:

$$\begin{array}{ccc} \frac{\partial H}{\partial t}-D_{H}\Delta H & = & \underset{\text{source}}{\underset{︸}{A_{H}}}+\underset{\text{proliferation}}{\underset{︸}{(\lambda_{HG}\frac{G}{G+K_{G}}+\lambda_{HT_{\beta }}\frac{T_{\beta }}{T_{\beta }+K_{T_{\beta }}})H}}-\underset{H\to H_{i}}{\underset{︸}{\lambda_{HH_{i}}V_{e}H}}-\underset{\text{death}}{\underset{︸}{\mu_{H}H}} \end{array}$$ (5)

The infected HSCs satisfy the equation

$$\begin{array}{ccc} \frac{\partial H_{i}}{\partial t}-D_{H}\Delta H_{i} & = & \underset{H\to H_{i}}{\underset{︸}{\lambda_{HH_{i}}V_{e}H}}-\underset{\text{death}}{\underset{︸}{\mu_{H}\left(1+\mu_{V_{iH}}V_{iH}\right)H_{i}}}, \end{array}$$ (6)

where the death rate of infected HSCs is increased due to the viral load *μ*_*V*_*iH*__ *V*_*iH*_.

**Equations for T cells density.** Th1 cells are activated from naive T cells (*T*_0_) by contact with M1 macrophages, under IL-12 environment [33]; this process is resisted by IL-10 and IL-13 [17, 73]. IL-2 enhances the proliferation of Th1 cells [17, 65]. Hence the equations for the densities of Th1 and Th2 cells are given as follows:

$$\begin{array}{ccc} \frac{\partial T_{1}}{\partial t}-D_{T}\Delta T_{1} & = & \underset{\text{activation}}{\underset{︸}{\left(\lambda_{T_{1}M_{1}}T_{0}\frac{M_{1}}{M_{1}+K_{M_{1}}}\frac{I_{12}}{I_{12}+K_{12}}\frac{1}{1+I_{10}/{\tilde{K}}_{10}}+\lambda_{T_{1}I_{2}}\frac{I_{2}}{I_{2}+K_{2}}T_{1}\right)\frac{1}{1+I_{13}/{\tilde{K}}_{13}}}}\\ & & -\underset{\text{death}}{\underset{︸}{\mu_{T_{1}}T_{1}}} \end{array}$$ (7)

*T*_0_ cells in the liver may also be activated into Th2 cells (*T*_2_) by contact with *M*_2_ in an *I*_4_ environment [36]. The activation of *T*_2_ is antagonized by *T*_1_ [36, 40, 41].

$$\begin{array}{ccc} \frac{\partial T_{2}}{\partial t}-D_{T}\Delta T_{2} & = & \underset{\text{activation}}{\underset{︸}{\lambda_{T_{2}M_{2}}T_{0}\frac{M_{2}}{M_{2}+K_{M_{2}}}\frac{I_{4}}{I_{4}+K_{4}}\frac{1}{1+T_{1}/{\tilde{K}}_{T_{1}}}}}-\underset{\text{death}}{\underset{︸}{\mu_{T_{2}}T_{2}}} \end{array}$$ (8)

**Equations for fibroblast (*f*) and myofibroblast (*m*) densities.** Hyaluronic acid (*H*_*A*_) enhances the proliferation of fibroblasts [44]. Fibroblasts are transformed into myofibroblasts by *G* and *T*_*β*_ [30, 46, 47, 74, 75]. Hence equations of *f* and *m* are:

$$\begin{array}{ccc} \frac{\partial f}{\partial t}-D_{f}\Delta f & = & \underset{\text{activation}}{\underset{︸}{\lambda_{fH_{A}}\frac{H_{A}}{H_{A}+K_{H_{A}}}f}}-\underset{f\to m}{\underset{︸}{(\lambda_{mfT_{\beta }}\frac{T_{\beta }}{T_{\beta }+K_{T_{\beta }}}+\lambda_{mfG}\frac{G}{G+K_{G}})f}}-\underset{\text{death}}{\underset{︸}{\mu_{f}f}} \end{array}$$ (9)

$$\begin{array}{ccc} \frac{\partial m}{\partial t}-D_{m}\Delta m & = & \underset{f\to m}{\underset{︸}{(\lambda_{mfT_{\beta }}\frac{T_{\beta }}{T_{\beta }+K_{T_{\beta }}}+\lambda_{mfG}\frac{G}{G+K_{G}})f}}-\underset{\text{death}}{\underset{︸}{\mu_{m}m}} \end{array}$$ (10)

**Equations for ECM (*ρ*) and scar (*S*) densities.** The ECM consists primarily of fibrillar collagens and elastins, but it includes also fibronectins, lamina and nitrogen that support the matrix network by connecting or linking collagens [49]. For simplicity we represent the ECM by the density of collagens. ECM is produced by *f*, *m* and by HSCs [47, 48]. The production of ECM by *m* is enhanced by *T*_*β*_ [47, 50]. We assume that MMP degrades the ECM at a rate proportional to *M*_*P*_ *ρ*. Hence the equation of *ρ* is as follows:

$$\begin{array}{ccc} \frac{d\rho }{dt} & = & \underset{\text{production}}{\underset{︸}{\lambda_{\rho f}f{\left(1-\frac{\rho }{\rho_{0}}\right)}^{+}+\lambda_{\rho m}\left(1+\lambda_{\rho T_{\beta }}\frac{T_{\beta }}{T_{\beta }+K_{T_{\beta }}}\right)m+\left(\lambda_{\rho H}H+\lambda_{\rho H_{i}}H_{i}\right)}}\\ & & -\underset{\rho \to S}{\underset{︸}{d_{\rho M_{P}}M_{P}\rho }}-\underset{\text{death}}{\underset{︸}{\mu_{\rho }\rho }}, \end{array}$$ (11)

where ${\left(1-\frac{\rho }{\rho_{0}}\right)}^{+}=1-\frac{\rho }{\rho_{0}}$ if *ρ* < *ρ*_0_, ${\left(1-\frac{\rho }{\rho_{0}}\right)}^{+}=0$ if *ρ* ≥ *ρ*_0_.

Fibrotic diseases are characterized by excessive scarring due to excessive production and deposition of ECM and disruption of normal healthy protein cross-linking. MMP disrupt collagen cross-linking and increases scarring in cases of excessive collagen concentrations [51]. Accordingly we model the growth of a scar as, in [51], by the formula

$$\begin{array}{ccc} S & = & \lambda_{S}{\left(\rho -\rho^{*}\right)}^{+}\left(1+\lambda_{SM_{P}}\frac{M_{P}}{M_{P}+K_{M_{P}}}\right), \end{array}$$ (12)

where ρ* is the concentration of collagen in normal healthy tissue.

**Equations for cytokines and other proteins.**

PDGF (*G*) is produced by *M*_2_ [31, 32]. Hence,

$$\begin{array}{ccc} \frac{\partial G}{\partial t}-D_{G}\Delta G & = & \underset{\text{production}}{\underset{︸}{\lambda_{GM_{2}}M_{2}+\lambda_{GM_{2i}}M_{2i}}}-\underset{\text{degradation}}{\underset{︸}{\mu_{G}G}} \end{array}$$ (13)

Hyaluronic acid (*H*_*A*_) is produced by HSCs and is degraded by sinusoidal epithelial cells [42, 43]. Hence *H*_*A*_ satisfies the following equation

$$\begin{array}{ccc} \frac{\partial H_{A}}{\partial t}-D_{H_{A}}\Delta H_{A} & = & \underset{\text{production}}{\underset{︸}{\lambda_{H_{A}H}H+\lambda_{H_{A}H_{i}}H_{i}}}-\underset{\text{degradation}}{\underset{︸}{\mu_{H_{A}}H_{A}}} \end{array}$$ (14)

where, for simplicity, the degradation of *H*_*A*_ is taken at a constant rate.

IL-1*β* is secreted by healthy and infected *M*_2_, and this process is inhibited by *I*_*α*_ and *I*_10_ [17, 18, 20, 24]. Hence,

$$\begin{array}{ccc} \frac{\partial I_{1\beta }}{\partial t}-D_{I_{1\beta }}\Delta I_{1\beta } & = & \underset{\text{production}}{\underset{︸}{\left(\lambda_{I_{1\beta }M_{2}}M_{2}+\lambda_{I_{1\beta }M_{2i}}M_{2i}\right)\frac{1}{1+I_{\alpha }/{\tilde{K}}_{\alpha }}\frac{1}{1+I_{10}/{\tilde{K}}_{10}}}}-\underset{\text{degradation}}{\underset{︸}{\mu_{I_{1\beta }}I_{1\beta }}} \end{array}$$ (15)

IL-2 is secreted by *T*_1_ [17]; hence,

$$\begin{array}{ccc} \frac{\partial I_{2}}{\partial t}-D_{I_{2}}\Delta I_{2} & = & \underset{\text{production}}{\underset{︸}{\lambda_{I_{2}T_{1}}T_{1}}}-\underset{\text{degradation}}{\underset{︸}{\mu_{I_{2}}I_{2}}} \end{array}$$ (16)

IL-4 is secreted by *T*_1_, *M*_2_ and *M*_2*i*_ [25, 26], so that the equation of *I*_4_ is given by

$$\begin{array}{ccc} \frac{\partial I_{4}}{\partial t}-D_{I_{4}}\Delta I_{4} & = & \underset{\text{production}}{\underset{︸}{\lambda_{I_{4}M_{2}}M_{2}+\lambda_{I_{4}M_{2i}}M_{2i}+\lambda_{I_{4}T_{2}}T_{2}}}-\underset{\text{degradation}}{\underset{︸}{\mu_{I_{4}}I_{4}}} \end{array}$$ (17)

IL-6 is produced by healthy and *M*_1_ macrophages, a process enhanced by TNF-*α* [17–19]. Hence,

$$\begin{array}{ccc} \frac{\partial I_{6}}{\partial t}-D_{I_{6}}\Delta I_{6} & = & \underset{\text{production}}{\underset{︸}{\left(\lambda_{I_{6}M_{1}}M_{1}+\lambda_{I_{6}M_{1i}}M_{1i}\right)\left(1+\frac{T_{\alpha }}{T_{\alpha }+K_{T_{\alpha }}}\right)}}-\underset{\text{degradation}}{\underset{︸}{\mu_{I_{6}}I_{6}}} \end{array}$$ (18)

IL-10 is produced by *M*_2_ and *M*_2*i*_ [17–20], and by *T*_2_ [27, 28]; so that

$$\begin{array}{ccc} \frac{\partial I_{10}}{\partial t}-D_{I_{10}}\Delta I_{10} & = & \underset{\text{production}}{\underset{︸}{\lambda_{I_{10}M_{2}}M_{2}+\lambda_{I_{10}M_{2i}}M_{2i}+\lambda_{I_{10}T_{2}}T_{2}}}-\underset{\text{degradation}}{\underset{︸}{\mu_{I_{10}}I_{10}}} \end{array}$$ (19)

IL-12 is produced by *M*_1_ and *M*_1*i*_, a process inhibited by IL-10 and IL-13 [17, 20, 21]. Hence

$$\begin{array}{ccc} \frac{\partial I_{12}}{\partial t}-D_{I_{12}}\Delta I_{12} & = & \underset{\text{production}}{\underset{︸}{\left(\lambda_{I_{12}M_{1}}M_{1}+\lambda_{I_{12}M_{1i}}M_{1i}\right)\frac{1}{1+I_{10}/{\tilde{K}}_{10}}\frac{1}{1+I_{13}/{\tilde{K}}_{13}}}}-\underset{\text{degradation}}{\underset{︸}{\mu_{I_{12}}I_{12}}} \end{array}$$ (20)

IL-13 is produced by *M*_2_ and *M*_2*i*_ [17–20], and by *T*_2_ [27–29]. Hence the equation of *I*_13_ is given as follows:

$$\begin{array}{c} \frac{\partial I_{13}}{\partial t}-D_{I_{13}}\Delta I_{13}=\underset{\text{production}}{\underset{︸}{\lambda_{I_{13}M_{2}}M_{2}+\lambda_{I_{13}M_{2i}}M_{2i}+\lambda_{I_{13}T_{2}}T_{2}}}-\underset{\text{degradation}}{\underset{︸}{\mu_{I_{13}}I_{13}}} \end{array}$$ (21)

IFN-*α* is produced by *T*_1_ and *T*_2_, a process resisted by IL-1*β* [34], so that

$$\begin{array}{c} \frac{\partial I_{\alpha }}{\partial t}-D_{I_{\alpha }}\Delta I_{\alpha }=\underset{\text{production}}{\underset{︸}{\left(\lambda_{I_{\alpha }T_{1}}T_{1}+\lambda_{I_{\alpha }T_{2}}T_{2}\right)\frac{1}{1+I_{1\beta }/{\tilde{K}}_{1\beta }}}}-\underset{\text{degradation}}{\underset{︸}{\mu_{I_{\alpha }}I_{\alpha }}} \end{array}$$ (22)

IFN-*γ* is secreted by *T*_1_ [17, 20, 35], a process resisted by IFN-*α* [23]. Hence IFN-*γ* satisfies the equation

$$\begin{array}{c} \frac{\partial I_{\gamma }}{\partial t}-D_{I_{\gamma }}\Delta I_{\gamma }=\underset{\text{production}}{\underset{︸}{\lambda_{I_{\gamma }T_{1}}\frac{1}{1+I_{\alpha }/{\tilde{K}}_{\alpha }}T_{1}}}-\underset{\text{degradation}}{\underset{︸}{\mu_{I_{\gamma }}I_{\gamma }}} \end{array}$$ (23)

TNF-*α* is secreted by *M*_1_, *M*_1*i*_ and *M*_2*i*_, a process resisted by IL-10 [22] and IL-13 [23]. Hence the equation of *T*_*α*_ is given by

$$\begin{array}{ccc} \frac{\partial T_{\alpha }}{\partial t}-D_{T_{\alpha }}\Delta T_{\alpha } & = & \underset{\text{production}}{\underset{︸}{\left(\lambda_{T_{\alpha }M_{1}}M_{1}+\lambda_{T_{\alpha }M_{1i}}M_{1i}+\lambda_{T_{\alpha }M_{2i}}M_{2i}\right)\frac{1}{1+I_{10}/{\tilde{K}}_{10}}\frac{1}{1+I_{13}/{\tilde{K}}_{13}}}}\\ & & -\underset{\text{degradation}}{\underset{︸}{\mu_{T_{\alpha }}T_{\alpha }}} \end{array}$$ (24)

TGF-*β* is produced by *M*_2_ and *M*_2*i*_, a process enhanced by IL-13 [30, 75]. Hence,

$$\begin{array}{ccc} \frac{\partial T_{\beta }}{\partial t}-D_{T_{\beta }}\Delta T_{\beta } & = & \underset{\text{production}}{\underset{︸}{\left(\lambda_{T_{\beta }M_{2}}M_{2}+\lambda_{T_{\beta }M_{2i}}M_{2i}\right)\left(1+\lambda_{T_{\beta }I_{13}}\frac{I_{13}}{I_{13}+K_{13}}\right)}}-\underset{\text{degradation}}{\underset{︸}{\mu_{T_{\beta }}T_{\beta }}} \end{array}$$ (25)

MMP and TIMP are produced by *M*_2_ and *M*_2*i*_ macrophages [31, 32], and depleted by binding to each other [73]. Hence the equations for *M*_*P*_ and *T*_*P*_ are given, respectively, as follows:

$$\begin{array}{c} \frac{\partial M_{P}}{\partial t}-D_{M_{P}}\Delta M_{P}=\underset{\text{production}}{\underset{︸}{\lambda_{M_{P}M_{2}}M_{2}+\lambda_{M_{P}M_{2i}}M_{2i}}}-\underset{\text{depletion}}{\underset{︸}{d_{M_{P}T_{P}}T_{P}M_{P}}}-\underset{\text{degradation}}{\underset{︸}{\mu_{M_{P}}M_{P}}} \end{array}$$ (26)

$$\begin{array}{c} \frac{\partial T_{P}}{\partial t}-D_{T_{P}}\Delta T_{P}=\underset{\text{production}}{\underset{︸}{\lambda_{T_{P}M_{2}}M_{2}+\lambda_{T_{P}M_{2i}}M_{2i}}}-\underset{\text{depletion}}{\underset{︸}{d_{T_{P}M_{P}}M_{P}T_{P}}}-\underset{\text{degradation}}{\underset{︸}{\mu_{T_{P}}T_{P}}} \end{array}$$ (27)

CCL3 is produced by infected *M*_1_ and *M*_2_ macrophages, as well as infected HSCs [45, 52, 53]. CCL3 is degraded naturally, but is also lost when internalized by *M*_1_ [64]. Hence *C*_3_ satisfies the equation:

$$\begin{array}{c} \frac{\partial C_{3}}{\partial t}-D_{C_{3}}\Delta C_{3}=\underset{\text{production}}{\underset{︸}{\lambda_{C_{3}H_{i}}H_{i}+\lambda_{C_{3}M_{1i}}M_{1i}+\lambda_{C_{3}M_{2i}}M_{2i}}}-\underset{\text{degradation}}{\underset{︸}{d_{C_{3}M_{1}}\frac{C_{3}}{C_{3}+K_{C_{3}}}M_{1}-\mu_{C_{3}}C_{3}}} \end{array}$$ (28)

**Equations for intracellular (*V*_*i*1_, *V*_*i*2_, *V*_*iH*_) and extracellular (*V*_*e*_) viruses.** In order to explain the dynamics of the viral loads, we introduce the following notations: *C* will denote any of the cells *M*_1_, *M*_2_ or *H*, and *C*_*i*_ will denote their infected states. *V*_*i*_ will denote any of the intracellular viral loads *V*_*i*1_, *V*_*i*2_ or *V*_*iH*_. We assume that the growth rate of *V*_*i*_ is proportional to *C*_*i*_, and is resisted by *I*_*α*_, *T*_*α*_, *T*_*β*_ and *I*_*γ*_, with the effect of *I*_*γ*_ being modulated by *I*_6_ [20, 35, 55], thus reducing the growth rate coefficient λ by a factor $\left(1+I_{\alpha }/{\tilde{K}}_{\alpha }\right)\left(1+T_{\alpha }/{\tilde{K}}_{T_{\alpha }}\right)\left(1+T_{\beta }/{\tilde{K}}_{T_{\beta }}\right)\left(1+T_{\gamma }/\left({\tilde{K}}_{\gamma }+I_{6}\right)\right)$. There is also an increase of *V*_*i*_ at a rate proportional to *CV*_*e*_ when external viruses are internalized by *C* cells [20]. We assume that the rate of this increase is λ_*CC*_*i*__ *N*_*V*_ *CV*_*e*_, where *N*_*V*_ is a dimensionality coefficient which is of order of magnitude of (mass of 1 virus)/(mass of 1 cell). We assume that when one infected cell, *C*_*i*_, dies at rate *μ*_*C*_*i*__ = *μ*_*C*_(1 + *μ*_*V*_*i*__ *V*_*i*_), *N* viruses are released. We conclude that the density of intracellular viruses in *C*_*i*_ satisfies the equation

$$\begin{array}{ccc} \frac{\partial V_{i}}{\partial t}-D_{C_{i}}\Delta V_{i} & = & \frac{\lambda C_{i}}{\left(1+I_{\alpha }/{\tilde{K}}_{\alpha }\right)\left(1+T_{\alpha }/{\tilde{K}}_{T_{\alpha }}\right)\left(1+T_{\beta }/{\tilde{K}}_{T_{\beta }}\right)\left(1+I_{\gamma }/\left({\tilde{K}}_{\gamma }+I_{6}\right)\right)}\\ & & +\lambda_{CC_{i}}N_{V}CV_{e}-\mu_{C}\left(1+\mu_{V_{i}}V_{i}\right)NV_{i}. \end{array}$$

Hence, we have the following equations for *V*_*i*1_, *V*_*i*2_, *V*_*iH*_ and *V*_*e*_:

$$\begin{array}{ccc} \frac{\partial V_{i1}}{\partial t}-D_{M}\Delta V_{i1} & = & \underset{\text{growth}\;\text{in}\;M_{1i}}{\underset{︸}{\frac{\lambda_{V_{i}M_{1i}}M_{1i}}{\left(1+I_{\alpha }/{\tilde{K}}_{\alpha }\right)\left(1+T_{\alpha }/{\tilde{K}}_{T_{\alpha }}\right)\left(1+T_{\beta }/{\tilde{K}}_{T_{\beta }}\right)\left(1+I_{\gamma }/\left({\tilde{K}}_{\gamma }+I_{6}\right)\right)\left(1+A/{\tilde{K}}_{A}\right)}}}\\ & & +\underset{V_{e}\to V_{i}}{\underset{︸}{\lambda_{M_{1}M_{1i}}N_{V}M_{1}V_{e}}}-\underset{\text{death}}{\underset{︸}{\mu_{M_{1}}\left(1+\mu_{V_{i1}}V_{i1}\right)NV_{i1}}} \end{array}$$ (29)

$$\begin{array}{ccc} \frac{\partial V_{i2}}{\partial t}-D_{M}\Delta V_{i2} & = & \underset{\text{growth}\;\text{in}\;M_{2i}}{\underset{︸}{\frac{\lambda_{V_{i}M_{2i}}M_{2i}}{\left(1+I_{\alpha }/{\tilde{K}}_{\alpha }\right)\left(1+T_{\alpha }/{\tilde{K}}_{T_{\alpha }}\right)\left(1+T_{\beta }/{\tilde{K}}_{T_{\beta }}\right)\left(1+I_{\gamma }/\left({\tilde{K}}_{\gamma }+I_{6}\right)\right)\left(1+A/{\tilde{K}}_{A}\right)}}}\\ & & +\underset{V_{e}\to V_{i}}{\underset{︸}{\lambda_{M_{2}M_{2i}}N_{V}M_{2}V_{e}}}-\underset{\text{death}}{\underset{︸}{\mu_{M_{2}}\left(1+\mu_{V_{i2}}V_{i2}\right)NV_{i2}}} \end{array}$$ (30)

$$\begin{array}{ccc} \frac{\partial V_{iH}}{\partial t}-D_{H}\Delta V_{iH} & = & \underset{\text{growth}\;\text{in}\;H_{i}}{\underset{︸}{\frac{\lambda_{V_{i}H_{i}}H_{i}}{\left(1+I_{\alpha }/{\tilde{K}}_{\alpha }\right)\left(1+T_{\alpha }/{\tilde{K}}_{T_{\alpha }}\right)\left(1+T_{\beta }/{\tilde{K}}_{T_{\beta }}\right)\left(1+I_{\gamma }/\left({\tilde{K}}_{\gamma }+I_{6}\right)\right)\left(1+A/{\tilde{K}}_{A}\right)}}}\\ & & +\underset{V_{e}\to V_{i}}{\underset{︸}{\lambda_{HH_{i}}N_{V}HV_{e}}}-\underset{\text{death}}{\underset{︸}{\mu_{H}\left(1+\mu_{V_{iH}}V_{iH}\right)NV_{iH}}} \end{array}$$ (31)

Note that the diffusion coefficients of intracellular viruses were taken the same as the diffusion coefficients of their hosts. Note also that we have included the factor $\frac{1}{1+A/{\tilde{K}}_{A}}$ in the first terms on the right-hand sides of Eqs (29)–(31). This factor accounts for the control of the viral load within infected cells by a drug, such as adefovir, that inhibits the growth of the virus. If the drug is not given, then *A* = 0 and the factor $\frac{1}{1+A/{\tilde{K}}_{A}}$ may be dropped.

*V*_*e*_ is increased when infected cells die and release the virus that they contained. There is a reduction in *V*_*e*_ when cells internalize extracellular viruses and become infected. Hence,

$$\begin{array}{ccc} \frac{\partial V_{e}}{\partial t}-D_{V_{e}}\Delta V_{e} & = & \underset{\text{released}\;\text{by}\;\text{infected}\;\text{cells}}{\underset{︸}{N[\mu_{M_{1}}\left(1+\mu_{V_{i1}}V_{i1}\right)V_{i1}+\mu_{M_{2}}\left(1+\mu_{V_{i2}}V_{i2}\right)V_{i2}+\mu_{H}\left(1+\mu_{V_{iH}}V_{iH}\right)V_{iH}]}}\\ & & -\underset{V_{e}\to V_{i}}{\underset{︸}{\left(\lambda_{M_{1}M_{1i}}M_{1}+\lambda_{M_{2}M_{2i}}M_{2}+\lambda_{HH_{i}}H\right)N_{V}V_{e}}}-\underset{\text{death}}{\underset{︸}{\mu_{V_{e}}V_{e}}}, \end{array}$$ (32)

**Boundary Conditions.** We simulate Model (1)–(32) in a rectangular domain *Ω*. We assume that the fibrosis occurs in *Ω* only, and hence we consider no-flux boundary conditions for all the variables.

**Initial Conditions.** We prescribe the following initial conditions in a unit of g/cm^3^:

Cells: *M*_1_(0) = 3.48 × 10^−2^, *M*_1*i*_(0) = 4.07 × 10^−3^, *M*_2_(0) = 4.02 × 10^−2^, *M*_2*i*_(0) = 4.86 × 10^−2^, *H*(0) = 4.97 × 10^−2^, *H*_*i*_(0) = 2.67 × 10^−4^, *T*_1_(0) = 9.44 × 10^−3^, *T*_2_(0) = 3.01 × 10^−2^, *f*(0) = 6.2 × 10^−3^, *m*(0) = 3.31 × 10^−4^;
ECM: *ρ*(0) = 2.7 × 10^−5^;
Cytokines and other proteins: *G*(0) = 1.81 × 10^−11^, *H*_*A*_(0) = 1.44 × 10^−6^, *I*_1*β*_(0) = 5.5 × 10^−10^, *I*_2_(0) = 1.47 × 10^−7^, *I*_4_(0) = 2.3 × 10^−10^, *I*_6_(0) = 1.29 × 10^−10^, *I*_10_(0) = 2.2 × 10^−9^, *I*_12_(0) = 4.19 × 10^−9^, *I*_13_(0) = 3.53 × 10^−10^, *I*_*α*_(0) = 7.42 × 10^−10^, *I*_*γ*_(0) = 1.77 × 10^−10^, *T*_*α*_(0) = 1.82 × 10^−10^, *T*_*β*_(0) = 6.22 × 10^−7^, *M*_*P*_(0) = 1.32 × 10^−5^, *T*_*P*_(0) = 3.86 × 10^−7^, *C*_3_(0) = 1.22 × 10^−10^;
HBV: *V*_*e*_(0) = 3.9 × 10^−12^, *V*_*i*1_(0) = *V*_*i*2_(0) = *V*_*iH*_(0) = 1.6 × 10^−9^.

However, we note that the simulation results with different initial conditions do not appreciably change after a few days.

## Results

All the computations are done using Python 2.7.6. The parameter values of the model equations are estimated in Section A in S1 File and are listed in Tables C–F in S1 File. The techniques used for the simulations are also described in Section B in S1 File.

**Fig 2:**
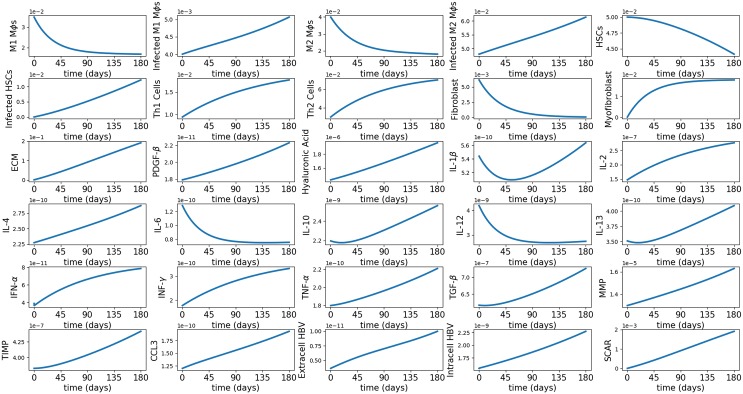
Simulation of all the average densities/concentrations of the variables for Model (1)–(28) over a period of 180 days.

Fig 2 is a simulation of the averages of the model variables for 180 days. We see that most of the variables are not in steady state, in agreement with the progressive state of HBV in chronic stage. The densities of uninfected phagocytic cells (*M*_1_, *M*_2_ and *H*) decrease while the densities of the infected phagocytic cells (*M*_1*i*_, *M*_2*i*_ and *H*_*i*_) increase. The increase in the densities of infected phagocytic cells results in an increase of the inflammation as seen in the profiles of IFN-*γ* and TNF-*α*, in agreement with chronic HBV progression. While *M*_2_ decreases and *M*_2*i*_ increases, the production term λ_*M*_*P*_*M*_2__ *M*_2_ + λ_*M*_*P*_*M*_2*i*__ *M*_2*i*_ of *M*_*P*_ increases, as seen in the increasing profile of *M*_*P*_. The simulations show that the densities of both Th1 cells and Th2 cells increase, while the Th2 cells are dominant. The increase in IL-10 and decrease in IL-12 suggest that the immune system is not strong enough to confront the infection. Cytokines produced by Th1 cells tend to reduce the load of intracellular viruses, while cytokines produced by Th2 cells work in the opposite direction. It is therefore interesting to note that, since *T*_2_ dominates *T*_1_, the viral loads (mostly represented by the intracellular HBV) are continuously increasing. Our model also shows that the density of fibroblasts decreases monotonically while the density of myofibroblasts increases, which enhances the production of ECM; the transition from fibroblast to myofibroblast is induced by PDGF and TGF-*β*, and it increases in time. The concentrations of MMP and TIMP also increase in time. The scar continues to grow, as a result of the increase in MMP and ECM.

We can also simulate the spatial variations of the variables, however we are interested, in this paper, only in the average profiles of the variables. One is tempted to use the simpler ODE model to compute averages. We note however that the diffusion coefficients of cytokines and extracellular viruses are several orders of magnitude larger than the diffusion coefficients of cells. For this reason the ODE system cannot adequately represent the spread of the infection, and hence, the averages of the variables. Indeed, in the simulation of the ODE system (not shown here) the infected macrophages decrease, rather than increase; healthy HSCs are increasing, rather than decreasing, and the intracellular and extracellular viruses decrease and stabilize, rather than monotonically increase, as in Fig 2.

## Treatment

In this section, we consider two drugs that are commonly used for the treatment of HBV, namely IFN-*α* and adefovir [5, 6, 12, 13]. Treatment with IFN-*α* in chronic HBV adult patient ranges between 16–52 weeks, while treatment with adefovir is continuous for 2–3 years [39, 76]. In oncology, it is found that in case of intermittent therapies in which the drug amount administered per unit time is constant, the response is significantly influenced by the amount of drug given and the period of time within which the drug is given [77–79]. Moreover, if negative side effects are ignored, then by doubling the amount of drug and correspondingly shortening the period of treatment (keeping the total amount of drug fixed fixed) we may get better results in reducing the infection [65]. Assuming that the total amount of each drug is fixed, we use the mathematical model to evaluate the efficacy of combination therapy with IFN-*α* and adefovir. We start the treatment after 90 days of control and administer the drugs for 90 days.

**Treatment with IFN-*α*.** In our model, injection of IFN-*α* is represented as a source term, *c*_*α*_(*t*), in Eq (22), which then takes the form

$$\begin{array}{c} \frac{\partial I_{\alpha }}{\partial t}-D_{I_{\alpha }}\Delta I_{\alpha }=\underset{\text{source}\;\text{term}}{\underset{︸}{c_{\alpha }\left(t\right)}}+\underset{\text{production}}{\underset{︸}{\lambda_{I_{\alpha }T_{1}}T_{1}+\lambda_{I_{\alpha }T_{2}}M_{2i}\frac{1}{1+I_{1\beta }/{\tilde{K}}_{1\beta }}}}-\underset{\text{degradation}}{\underset{︸}{\mu_{I_{\alpha }}I_{\alpha }}}. \end{array}$$ (33)

**Treatment with adefovir (*A*).** We denote the source of the drug by *c*_*A*_(*t*). The drug is diminished by absorption by infected cells, and by natural degradation. Hence, the equation of adefovir, *A*, is given by

$$\begin{array}{c} \frac{\partial A}{\partial t}-D_{A}\Delta A=\underset{\text{source}}{\underset{︸}{c_{A}\left(t\right)}}-\underset{\text{absorption}}{\underset{︸}{\left(\lambda_{M_{1i}A}M_{1i}+\lambda_{M_{2i}A}M_{2i}+\lambda_{H_{i}A}H_{i}\right)\frac{A}{A+K_{A}}}}-\underset{\text{degradation}}{\underset{︸}{\mu_{A}A}}. \end{array}$$ (34)

**Combination therapy with IFN-*α* and adefovir.** Combination therapy with IFN-*α* and adefovir is modeled by using Eqs (33) and (34) together with System (1)–(32). Adefovir is given daily in one or two tablets [80], and its effective time is about 10 hours [81]. For simplicity, we assume a daily average constant level *c*_*A*_ taking

$$\begin{array}{c} c_{A}=\left\{ \begin{array}{cc} 10^{-4}\;\text{g}/{\text{cm}}^{3}{\text{d}}^{-1}, & 90<t<180;\\ 0, & \text{elsewhere}. \end{array}\right. \end{array}$$ (35)

IFN-*α* is given in shots three times a week, or in slow injection, as pegylated IFN-*α*, once a week [82]. We assume that it is given once a week at level *c*_*α*_, so that after *t* days its level reduces to

$$\begin{array}{c} c_{\alpha }e^{-\sigma t},\;\text{for}\;\text{some}\;\sigma >0, \end{array}$$

and at the end of the week (*t* = 7 days) its level is negligible, taking

$$\begin{array}{c} c_{\alpha }e^{-7\sigma }=\frac{1}{X}c_{\alpha },\;X=e^{7}, \end{array}$$

so that *σ* = 1. The average level during the week is

$$\begin{array}{c} \frac{1}{7}\int_{0}^{7}c_{\alpha }e^{-t}dt=\frac{c_{\alpha }}{7}\left(1-e^{-7}\right)∼\frac{c_{\alpha }}{7}. \end{array}$$

We wish to simplify the simulations by replacing the intermittent treatment of injections at level 7*c*_*α*_ by a constant level of the drug at the level *c*_*α*_, that is, by

$$\begin{array}{c} c_{\alpha }\left(t\right)=\left\{ \begin{array}{cc} 10^{-7}\text{g}/{\text{cm}}^{3}{\text{d}}^{-1}, & 90<t<180;\\ 0, & \text{elsewhere}. \end{array}\right. \end{array}$$ (36)

**Fig 3:**
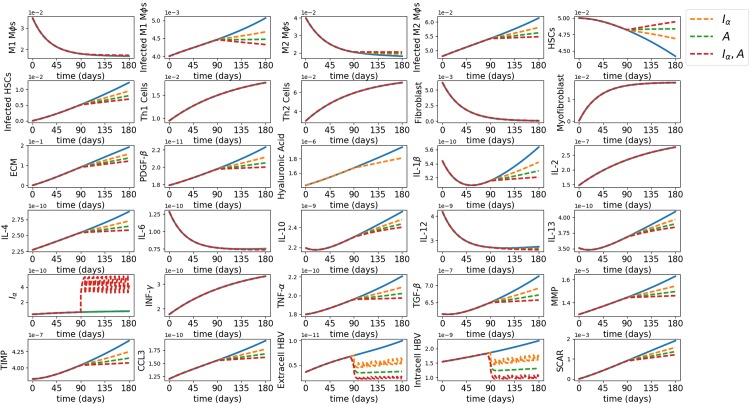
Monotherapy and combination therapy with intermittent IFN-*α* and continuous adefovir. The horizontal axes scale the time in days and the vertical axes scale the average densities in g/cm^3^ for all the variables for the first 180 days since the start of the disease, in control case (blue) and with treatment (IFN-*α*–orange, adefovir–blue and combination–red).

**Fig 4:**
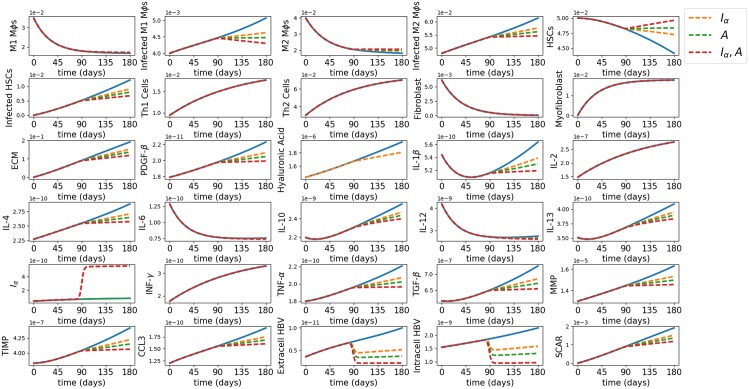
Monotherapy and combination therapy with IFN-*α* and adefovir. The horizontal axes scale the time in days and the vertical axes scale the average densities in g/cm^3^ for all the variables for the first 180 days since the start of the disease, in control case (blue) and with treatment (IFN-*α*–orange, adefovir–blue and combination–red).

In order to justify this simplification, we simulated in Figs 3 and 4 the average densities of all the variables with treatment (IFN-*α*–orange, adefovir–green and combination–red) and without treatment when the drugs are given intermittently (Fig 3) and continuously (Fig 4).

We see that the profiles of the virus and scar are almost the same under Figs 3 and 4. Hence, for simplicity, we shall use the scheme for Fig 4, that is, Eqs (35) and (36), to compute the efficacy and synergy maps.

**Therapy with continuous drug delivery.** From Fig 4, we see that IFN-*α* reduces the viral load by 30.36%, which agrees with reported clinical results by Paul and Han [83]; adefovir reduces by 42.28% and the combination reduces by 57.66%. The reduction in the scar is 20.41% by IFN-*α*, 28.24% by adefovir, and 37.8% by the combination, although the scar still keeps increasing. We also see that the drugs (in monotherapy and in combination therapy) effectively reduce the inflammation by considerably reducing the densities of infected phagocytic cells (*M*_1*i*_, *M*_2*i*_ and *H*_*i*_), and the pro-inflammatory cytokine TNF-*α*. The pro-inflammatory cytokines IL-6 and IL-12 already have decreasing profiles in the control case, making the effect of drugs on them less significant. We observe in Fig 4 that the drugs have little (or no) effect on the profiles of Th1 and Th2 cells, and hence also on IFN-*γ* and IL-2 which are secreted by T cells.

We proceed to consider the effect of therapy for a range of doses of adefovir and IFN-*α*. We denote by *S*_*X*_(180) and *V*_*i*,*X*_(180) the densities of *S* and *V*_*i*_, respectively, at day 180 under administration of drug *X*, where *X* is either adefovir alone, or IFN-*α* alone, or their combination. We denote by *S*(180) and *V*_*i*_(180) the densities of *S* and *V*_*i*_, respectively, at day 180 in the control case (no drugs), and define two efficacy functions:

$$\begin{array}{ccc} E_{S}\left(X\right) & = & \frac{S\left(180\right)-S_{X}\left(180\right)}{S(180)}\times \left(100\%\right)\\ E_{V_{i}}\left(X\right) & = & \frac{V_{i}\left(180\right)-V_{i,X}\left(180\right)}{V_{i}\left(180\right)}\times \left(100\%\right). \end{array}$$ (37)

**Fig 5:**
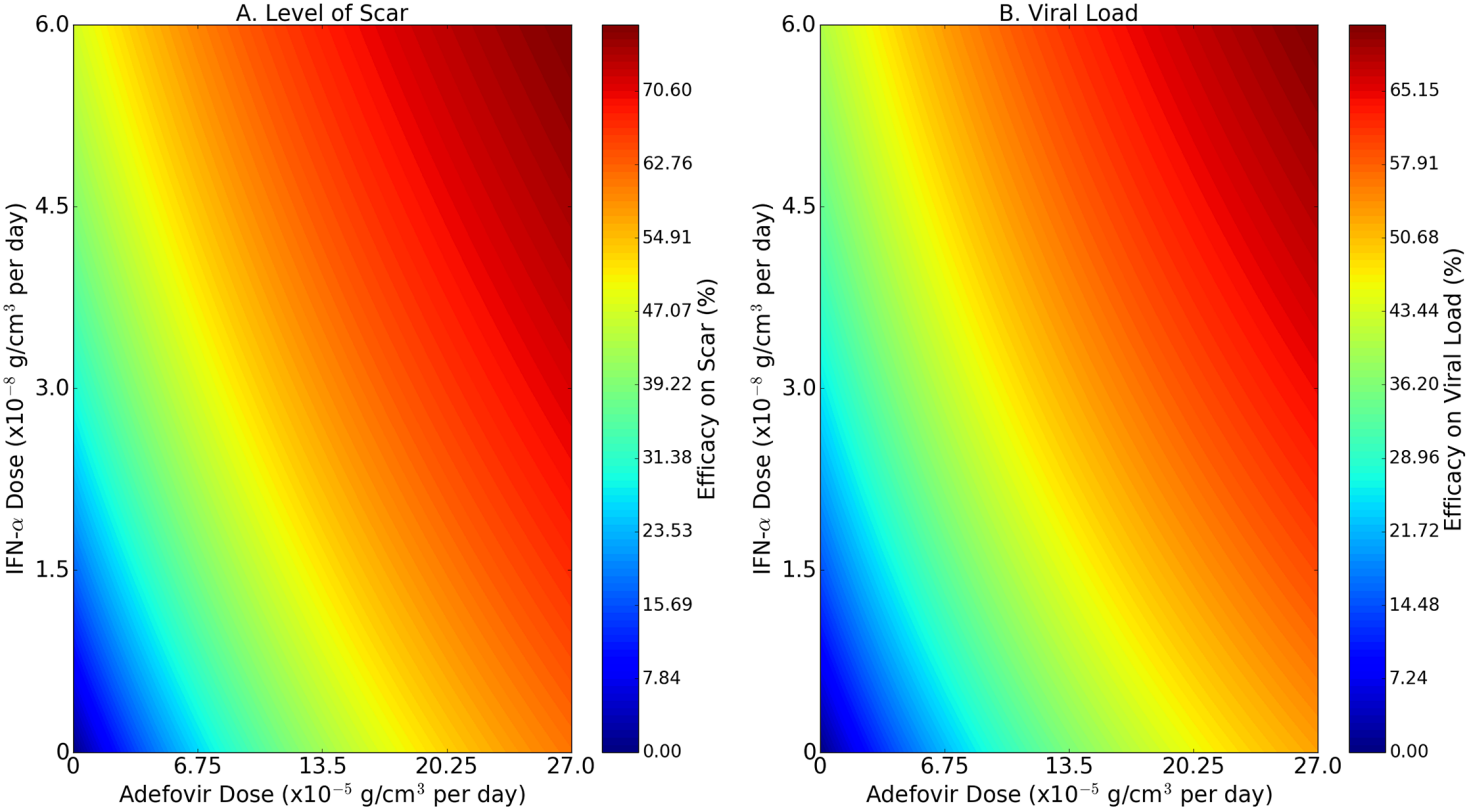
Efficacy map in combination therapy with IFN-*α* and adefovir. The horizontal axes scale the fractions for the dose of IFN-*α* and the vertical axes scale the fractions for the dose of adefovir. The color maps represent the efficacies of reduction in the level of scar density (A) and the viral load (B) at day 180 of infection.

Fig 5A is an efficacy map for the scar density under the combined therapy with *X* = (*c*_*A*_, *c*_*α*_), and Fig 5B is the corresponding efficacy map for the viral load *V*_*i*_; the color columns colors show the efficacy values.

Both IFN-*α* and adefovir can have severe side-effects when administered alone. These side-effects could be aggravated if both drugs are given simultaneously with the same doses as in monotherapy. Thus, there is the need to find a strategy for combination therapy that maximizes the efficacy while reducing the sides effects. The dose-response relationship of the amount of drugs to the severity of negative side-effects has been extensively studied [84–86]: at higher doses, undesired side-effects appear and grow stronger with increases in the dose.

We compare treatment of combination therapy (*c*_*α*_, *c*_*A*_) with monotherapy *I*_*α*_ and monotherapy *A*. For monotherapy *I*_*α*_, we take (1 + *θ*_*I*_*α*__)*c*_*α*_, and for monotherapy *A* we take (1 + *θ*_*A*_)*c*_*A*_, with *θ*_*I*_*α*__ > 0, *θ*_*A*_ > 0, to reflect the highest toxicity expected when combining the two drugs. If *E*_*S*_(*c*_*α*_, *c*_*A*_) is larger than both *E*_*S*_((1 + *θ*_*I*_*α*__)*c*_*α*_, 0) and *E*_*S*_(0, (1 + *θ*_*A*_)*c*_*A*_), then we say that the ‘synergy’ in scar density reduction for the combination (*c*_*α*_, *c*_*A*_) is positive, and otherwise, we say that the synergy is negative [87]. Similarly, if *E*_*V*_*i*__(*c*_*α*_, *c*_*A*_) is larger than both *E*_*V*_*i*__((1 + *θ*_*I*_*α*__)*c*_*α*_, 0) and *E*_*V*_*i*__(0, (1 + *θ*_*A*_)*c*_*A*_), then we say that the synergy in viral load reduction for the combination (*c*_*α*_, *c*_*A*_) is positive, and otherwise, we say that the synergy is negative [87]. More generally, we define the synergy in scar density reduction *σ*_*S*_ = *σ*_*S*_(*c*_*α*_, *c*_*A*_) by the formula (as in [87]):

$$\begin{array}{c} \sigma_{S}\left(c_{\alpha },c_{A}\right)=\frac{E_{S}\left(c_{\alpha },c_{A}\right)}{\max\{E_{S}\left(\left(1+\theta_{I_{\alpha }}\right)c_{\alpha },0\right),E_{S}\left(0,\left(1+\theta_{A}\right)c_{A}\right)\}}-1. \end{array}$$ (38)

Then *σ*_*S*_(*c*_*α*_, *c*_*A*_)>0 (positive synergy) if the combination (*c*_*α*_, *c*_*A*_) reduces the level of scar density more than either one of the single agents (1 + *θ*_*I*_*α*__)*c*_*α*_ or (1 + *θ*_*A*_)*c*_*A*_. Negative synergy occurs in the reverse case where instead of a combination therapy with (*c*_*α*_, *c*_*A*_) we achieve better reduction of the level of scar density if we apply only one drug, (1 + *θ*_*I*_*α*__)*c*_*α*_ or (1 + *θ*_*A*_)*c*_*A*_. Similarly we define the synergy in reducing the viral load by the formula:

$$\begin{array}{c} \sigma_{V_{i}}\left(c_{\alpha },c_{A}\right)=\frac{E_{V_{i}}\left(c_{\alpha },c_{A}\right)}{\max\{E_{V_{i}}\left(\left(1+\theta_{I_{\alpha }}\right)c_{\alpha },0\right),E_{V_{i}}\left(0,\left(1+\theta_{A}\right)c_{A}\right)\}}-1. \end{array}$$ (39)

There are negative side-effects such as lactic acidosis in the case of treatment with adefovir, and serious depression in the case of IFN-*α* [88]. Since the mechanisms of these side-effects are not well understood, and since it is difficult to evaluate and compare the severity of these side-effects, we take for definiteness *θ*_*I*_*α*__ = *θ*_*A*_ = 2; this choice is arbitrary and could be made more precise as more clinical data become available that will inform on the side-effects associated with IFN-*α* and adefovir, separately or in combination.

**Fig 6:**
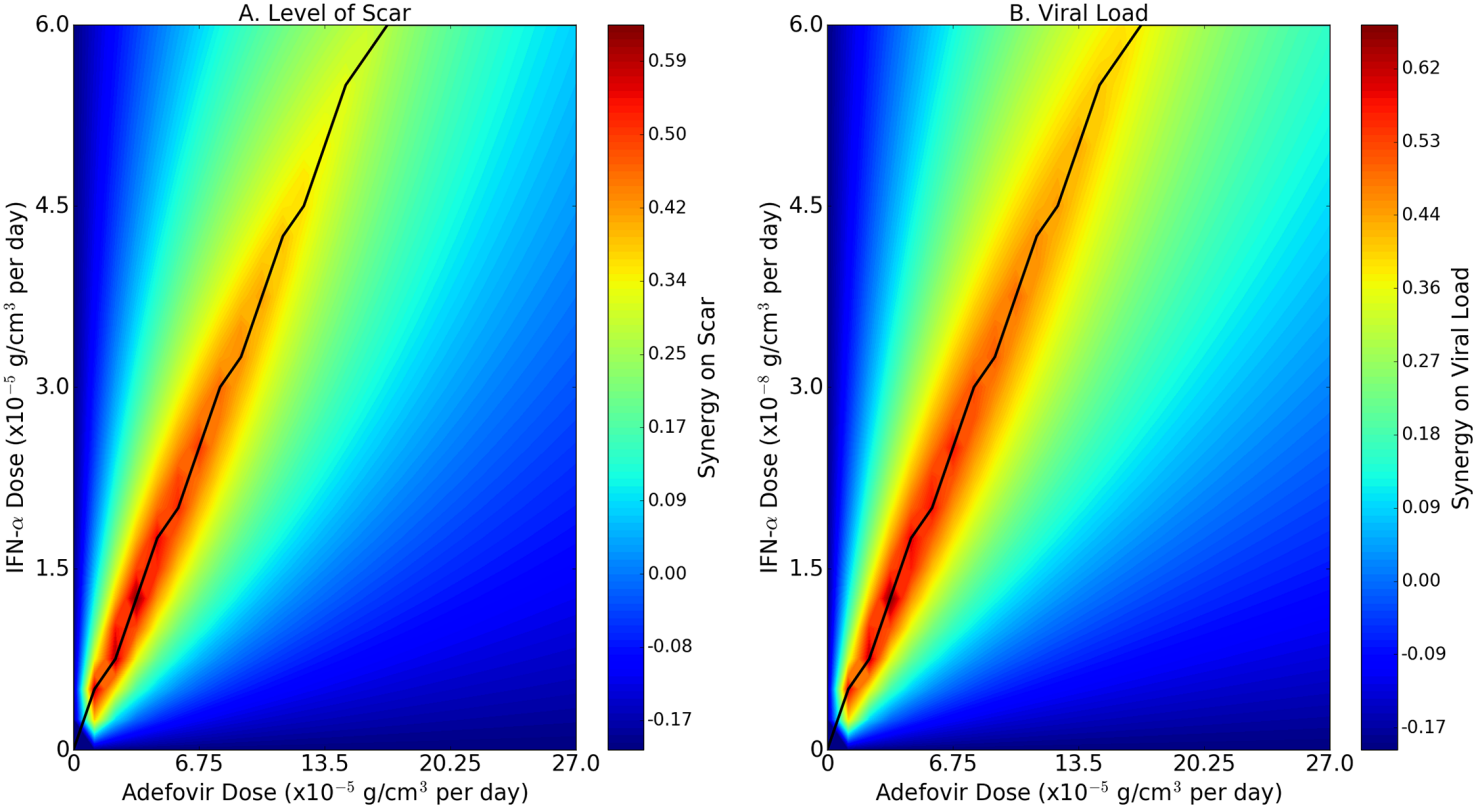
Synergy map for drugs IFN-*α* and adefovir in HBV. The horizontal axes scale the dose of IFN-*α* and the vertical axes scale the dose of adefovir. The color maps represent the synergies between the two drugs IFN-*α* and adefovir in reducing the level of scar density (A) and the viral load (B) at day 180 of infection.

Fig 6 shows the ‘synergy map’ associated with Model (1)–(32) where we compute the values for the synergy as in Eq (38) related with the drugs IFN-*α* (*I*_*α*_) and adefovir (*A*). The color columns assign a “synergy number” (SN) to any level of adefovir and IFN-*α*; SN varies from −0.16 to 0.64 for the level of scar density, and from −0.15 to 0.69 for the viral load. Given any level of *c*_*α*_, as *c*_*A*_ initially increases so does the SN, until *c*_*A*_ reaches a level $c_{A}^{*}$ which depends on *c*_*α*_; i.e., $c_{A}^{*}=c_{A}\left(c_{\alpha }\right)$. Thereafter, the SN begins to decrease as *c*_*A*_ increases. The biological explanation of the color patterns in the synergy maps can be traced to the fact that the virus proliferation is inhibited by the product $\left(1+I_{\alpha }/{\tilde{K}}_{\alpha }\right)\left(1+A/{\tilde{K}}_{A}\right)$, as seen in Eqs (29)–(31). This product is highly correlated, after scaling, to the product (1 + *c*_*α*_)(1 + *c*_*A*_). Viewing this product as representing the efficacy of the combined therapy, the resulting expression for the synergy is then a function of the following type:

$$\begin{array}{c} f\left(c_{\alpha },c_{A}\right)=\frac{\left(1+c_{\alpha }\right)\left(1+c_{A}\right)}{\max(1+3c_{\alpha },1+3c_{A})}-\text{constant}. \end{array}$$

We now observe that *f*(*c*_*α*_, *c*_*A*_) is an increasing function in *c*_*A*_ if *c*_*A*_ < *c*_*α*_ and a decreasing function in *c*_*A*_ if *c*_*A*_ > *c*_*α*_, and similarly, *f*(*c*_*α*_, *c*_*A*_) is increasing in *c*_*α*_ if *c*_*α*_ < *c*_*A*_ and is decreasing in *c*_*α*_ if *c*_*α*_ > *c*_*A*_; and this behavior is reflected in the synergy maps. In both synergy maps Fig 6A and 6B, SN is negative when *c*_*A*_ < 1.25 × 10^−5^ g/cm^3^ per day, or *c*_*α*_ < 0.15 × 10^−8^ g/cm^3^ per day. Negative synergy indicates that it is more beneficial to use monotherapy with either 3*c*_*α*_ or 3*c*_*A*_ instead of combination therapy with (*c*_*α*_, *c*_*A*_). However this conclusion does not take into account negative side-effects.

## Discussion

Hepatitis B virus (HBV) infection is one of the most prevalent infectious diseases associated with human liver. Despite the availability of a vaccine, HBV remains a global health problem, which affects more than 350 million people annually, with 600, 000 deaths resulting from HBV–related liver diseases. One of the hallmarks of the disease is the development of liver fibrosis. Anti–HBV drugs currently in use include adefovir, which is anti–viral therapy, and IFN-*α*, which is decreases viremia, inflammation and liver fibrosis. The treatment with each of these drugs typically extends over a period of many months, and incurs severe negative side-effects; kidney damage, low amount of phosphate in the blood, stroke and pancreatitis for adefovir, brain disorder, heart attack and lung fibrosis for IFN-*α*.

In the present paper, we considered the effect of these two drugs on slowing the growth of liver fibrosis associated with HBV. With the aim of using the least amount of drugs, we asked the following question: Is a combination of adefovir and IFN-*α* at a certain level more effective in reducing the fibrotic scar than a monotherapy with either adefovir or IFN-*α* at an ‘appropriate’ increased level? If so, we say that the combination therapy is *synergetic*. We developed a mathematical model by a system of PDEs and used the model to address this question. The model includes cells and cytokines that significantly affect the fibrosis of the liver in HBV. Some of the parameters were taken from the literature, while others were estimated under some assumptions; simulations of the model validated these assumptions.

The main result of the paper is the “synergy maps” in Fig 6. The color columns assign a “synergy numbers” (SN) to any level of adefovir (*c*_*A*_) and IFN-*α* (*c*_*α*_). Given any level of *c*_*α*_, as *c*_*A*_ initially increases so does each SN, until *c*_*A*_ reaches a level $c_{A}^{*}$ which depends on *c*_*α*_; i.e., $c_{\alpha }^{*}=c_{\alpha }\left(c_{A}\right)$. Thereafter, the SN begins to decrease as *A* increases. We suggest that the dosage of IFN-*α* should be related to adefovir so that the proportion should lie as closed as possible to both solid curves in Fig 6.

In Fig 6, negative synergy means that it is more beneficial to treat the patient with either IFN-*α* or adefovir as a single agent, rather than with combination of the two drugs. In the definition of efficacy we compared treatment of combination therapy (*c*_*α*_, *c*_*A*_) to monotherapy with either (1 + *θ*_*I*_*α*__)*c*_*α*_ or (1 + *θ*_*A*_)*c*_*A*_, where we took *θ*_*I*_*α*__ = *θ*_*A*_ = 2. This choice was quite arbitrary. If it turns out that the negative side-effects in the combined therapy are much more severe than the negative side-effects of a monotherapy with either IFN-*α* and adefovir, then it would be more appropriate to increase the values of *θ*_*A*_ and *θ*_*I*_*α*__ in the synergy map. On the other hand, if the negative side-effects from the combination are not significantly more severe than the negative side-effects from either one of the two drugs, then it would be appropriate to decrease the chosen values of *θ*_*A*_ and *θ*_*I*_*α*__ in the synergy map. A meta-analysis for the comparison of efficacy in the combination IFN-*α*-adefovir versus IFN-*α* in monotherapy is done in [89]. In this work that does not include the effect of drug toxicity, the authors show that the efficacy of IFN-*α* plus adefovir combination therapy is superior to IFN-*α* monotherapy. However, combined clinical data on efficacy and toxicity are quite limited at this time. Hence, our model should be viewed as setting up a computational framework, to address the question of optimal efficacy in combination therapy with IFN-*α* and adefovir.

## Supporting information

S1 File Supporting information—Chronic hepatitis B virus and liver fibrosis: A mathematical model. Parameter estimates (Section A in S1 File), parameter values (Tables C–F in S1 File) and numerical methods used (Section B in S1 File).
(PDF)

## References

[1] Inuzuka T, Takahashi K, Chiba T, Marusawa H. Mouse models of hepatitis B virus infection comprising host-virus immunologic interactions. Pathogens. 2014;3:377–389. doi: 10.3390/pathogens3020377 25437805 10.3390/pathogens3020377 PMC4243451

[2] Ott JJ, Stevens GA, Groeger J, Wiersma S. Global epidemiology of hepatitis B virus infection: new estimates of age-specific HBsAg seroprevalence and endemicity. Vaccine. 2012;30(12):2212–2219. doi: 10.1016/j.vaccine.2011.12.116 22273662 10.1016/j.vaccine.2011.12.116

[3] Schweitzer A, Horn J, Mikolajczyk RT, et al Estimations of worldwide prevalence of chronic hepatitis B virus infection: a systematic review of data published between 1965 and 2013. Lancet. 2015 10;386(10003):1546–1555. doi: 10.1016/S0140-6736(15)61412-X 26231459 10.1016/S0140-6736(15)61412-X

[4] Tzeng H, Tsai H, Liao H, Chen C, Chen P, Hsu P. Tumor necrosis factor-alpha induced by hepatitis B virus core mediating the immune response for hepatitis B viral clearance in mice model. PLoS ONE. 2014 7;9(7):1–8. doi: 10.1371/journal.pone.0103008 10.1371/journal.pone.0103008 PMC4105421 25047809

[5] Song J, Zhou Y, Li S, Wang B, Zheng X, Wu J, et al Susceptibility of different hepatitis B virus isolates to interferon-alpha in a mouse model based on hydrodynamic injection. PLoS ONE. 2014 3;9(3):1–9. doi: 10.1371/journal.pone.0090977 10.1371/journal.pone.0090977 PMC3950299 24618716

[6] Byrnes AA, Li DY, Park K, Thompson D, Mocilnikar C, Mohan P, et al Modulation of the IL-12/IFN-*γ* axis by IFN-*α* therapy for hepatitis C. J Leuc Biol. 2007 3;81:825–834. doi: 10.1189/jlb.1006622 10.1189/jlb.1006622 17148690

[7] Asselah T, Lada O, Roucani M, Martinot M, Boyer N, Marcellin P. Interferon therapy for chronic hepatitis B. Clin Liver Dis. 2007;11:839–849. doi: 10.1016/j.cld.2007.08.010 17981231 10.1016/j.cld.2007.08.010

[8] Liu Y, Wu T, Sun N, Wang G, Yuan J, Dai Y, et al Combination therapy with pegylated interferon alpha-2b and adfovir dipivoxil in HBeAg-positive chronic heaptitis B versus interferon alone: a prospective, randomized study. J Huazhong Univ Sci Technol. 2014;34(4):542–547. doi: 10.1007/s11596-014-1312-2 10.1007/s11596-014-1312-2 25135724

[9] Loggi E, Vitale G, Conti F, Bernardi M, Andreone P. Chronic hepatitis B: Are we close to a cure? Digestive and Liver Disease. 2015 10;47(10):836–841. doi: 10.1016/j.dld.2015.05.019 26138799 10.1016/j.dld.2015.05.019

[10] Ebert G, Preston S, Allison C, Cooney J, Toe JG, Stutz MD, et al Cellular inhibitor of apoptosis proteins prevent cleatance of hepatitis B virus. PNAS. 2015 5 5;112(18):5797–5802. doi: 10.1073/pnas.1502390112 25902529 10.1073/pnas.1502390112 PMC4426461

[11] Ebert G, Allison C, Preston S, Cooney J, Toe JG, Stutz MD, et al Eliminating hepatitis B by antagonizing cellular inhibitors of apoptosis. PNAS. 2015 5 5;112(18):5803–5808. doi: 10.1073/pnas.1502400112 25902530 10.1073/pnas.1502400112 PMC4426438

[12] Manolakopoulos S, Bethanis S, Koutsounas S, Goulis J, Vlachogiannakos J, Christias E, et al Long-term therapy with adefovir dipivoxil in hepatitis B e antigen-negative patients developing resistance to lamivudine. Aliment Pharmacol Ther. 2008 2;27(3):266–273. doi: 10.1111/j.1365-2036.2007.03567.x 17988233 10.1111/j.1365-2036.2007.03567.x

[13] Marcellin P, Chang TT, Lim SG, Sievert MJTW, Shiffman ML, Jeffers L, et al Adefovir dipivoxil for the treatment of hepatitis B e antigen-positive chronic hepatitis B. N Engl J Med. 2003 2;348(9):808–816. doi: 10.1056/NEJMoa020681 12606735 10.1056/NEJMoa020681

[14] WedMD, LLC. List interferon alfa-2b injection side effects by likelihood and severity. Side Effects. 2017;.

[15] WedMD, LLC. List adefovir side effects by likelihood and severity. Side Effects. 2017;.

[16] Bility MT, Chiang L, Zhang Z, Luan Y, Li F, Chi L, et al Hepatitis B Virus Infection and Immunopathogenesis in a Humanized Mouse Model: Induction of Human-Specific Liver Fibrosis and M2–Like Macrophages. PLOS Pathogens. 2014 3;10(3):1–14. doi: 10.1371/journal.ppat.1004032 10.1371/journal.ppat.1004032 PMC3961374 24651854

[17] Akdis M, Burgler S, Crameri R, Eiwegger T, Fujita H, Gomez E, et al Interleukins, from 1 to 37, and interferon-*γ*: Receptors, functions, and roles in diseases. J Allergy Clin Immunol. 2011 3;(127):701–721. doi: 10.1016/j.jaci.2010.11.050 21377040 10.1016/j.jaci.2010.11.050

[18] Boltjes A, Movita D, Boonstra A, Woltman AM. The role of Kupffer cells in hepatitis B and hepatitis C virus infections. Journal of Hepatology. 2014;61:660–671. doi: 10.1016/j.jhep.2014.04.026 24798624 10.1016/j.jhep.2014.04.026

[19] Liu H, Zhang X. Innate immune recognition of hepatitis B virus. World J Gastroenterol. 2015;7(21):2319–2322. 10.4254/wjh.v7.i21.2319 PMC4577638 26413220

[20] Phillips S, Chokshi S, Riva A, Evans A, Williams R, Naoumov NV. CD8^+^ T cell control of hepatitis B virus replication: Direct comparison between cytolytic and noncytolytic functions. J Immunol. 2010;184:287–295. doi: 10.4049/jimmunol.0902761 19949099 10.4049/jimmunol.0902761

[21] Poovorawan K, Tangkijvanich P, Chirathaworn C, Wisedopas N, Treeprasertsuk S, Komolmit P, et al Circulating cytokines and histological liver damage in chronic hepatitis B infection. Hepatitis Research and Treatment. 2013;2013(757246):1–7. doi: 10.1155/2013/757246 10.1155/2013/757246 PMC3833406 24288603

[22] Oswald IP, Wynn TA, Sher A, James SL. Interleukin 10 inhibits macrophage microbicidal activity by blocking the endogenous production of tumor necrosis factor alpha required as a costimulatory factor for interferon gamma-induced activation. Proc Natl Acad Sci USA. 1992;89(18):8676–8680. doi: 10.1073/pnas.89.18.8676 1528880 10.1073/pnas.89.18.8676 PMC49983

[23] Mayer-Baker KD, Andrade BB, otand SD, Amaral EP, Barber DL. Host-directed therapy of tuberculosis based on interleukin-1 and type I interferon crosstalk. Nature. 2014;511(7507):99–103. doi: 10.1038/nature13489 24990750 10.1038/nature13489 PMC4809146

[24] Senoo H. Structure and function of hepatic stellate cells. Med Electron Microsc. 2004 3;37(1):3–15. doi: 10.1007/s00795-003-0230-3 15057600 10.1007/s00795-003-0230-3

[25] Buttner C, Skupin A, Reimann T, Rieber EP, Unteregger G, Geyer P, et al Local production of interleukin-4 during radiation-induced pneumonitis and pulmonary fibrosis in rats: macrophages as a prominent source of interleukin-4. Am J Respir Cell Mol Biol. 1997;17(3):315–325. doi: 10.1165/ajrcmb.17.3.2279 9308918 10.1165/ajrcmb.17.3.2279

[26] Pouliot P, Turmel V, Gelinas E, Laviolette M, Bissonette EY. Interleukin-4 production by human alveolar macrophages. Clin Exp Allergy. 2005;35(6):804–810. doi: 10.1111/j.1365-2222.2005.02246.x 15969673 10.1111/j.1365-2222.2005.02246.x

[27] Jiang Y, Ma Z, Xin G, Yan H, Li W, Xu H, et al Th1 and Th2 Immune Response in Chronic Hepatitis B Patients during a Long-Term Treatment with Adefovir Dipivoxil. Mediators Inflamma. 2010;2010:1–10. doi: 10.1155/2010/143026 10.1155/2010/143026 PMC2994066 21127728

[28] Viallard JF, Pellegrin JL, Schaeverbeke T, Dehais J, Longy-Boursier M, Ragnaud JM, et al Th1 (IL-2, interferon-gamma (IFN-*γ*)) and Th2 (IL-10, IL-4) cytokine production by peripheral blood mononuclear cells (PBMC) from patients with systemic lupus erythematosus (SLE). Clin Exp Immunol. 1999 1;115(1):189–195. doi: 10.1046/j.1365-2249.1999.00766.x 9933441 10.1046/j.1365-2249.1999.00766.x PMC1905189

[29] Venkayya R, Lam M, Willkom M, Grunig G, Corry DB, Erle DJ. The Th2 lymphocyte products IL-4 and IL-13 rapidlyinduce airway hyperresponsiveness through direct effects on resident airway cells. Am J Respir Cell Mol Biol. 2002 10;26(2):202–208. doi: 10.1165/ajrcmb.26.2.4600 11804871 10.1165/ajrcmb.26.2.4600

[30] Fichtner-Feigl S, Strober W, Kawakami K, Puri RK, Kitani A. IL-13 signaling through the IL-13alpha2 receptor is involved in induction of TGF-beta1 production and fibrosis. Nat Med. 2006;12:99–106. doi: 10.1038/nm1332 16327802 10.1038/nm1332

[31] Vernon MA, Mylonas KJ, Hughes J. Macrophages and renal fibrosis. Semin Nephrol. 2010;30:302–317. doi: 10.1016/j.semnephrol.2010.03.004 20620674 10.1016/j.semnephrol.2010.03.004

[32] Zhao H, Dong Y, Tian X, Tan TK, Liu Z, Zhao Y, et al Matrix metalloproteinases contribute to kidney fibrosis in chronic kidney diseases. World J Nephrol. 2013;2:84–89. doi: 10.5527/wjn.v2.i3.84 24255890 10.5527/wjn.v2.i3.84 PMC3832915

[33] Janeway CA, Travers P, Walport M, Schlomchik MJ. Immunobiology: The Immune System in Health and Disease. 2nd ed.; 2001.

[34] Klimpel GR, Infante AJ, Patterson J, Hess CB, Asuncion M. Virus-induced interferon alpha/beta (IFN-alpha/beta) production by T cells and by Th1 and Th2 helper T cell clones: a study of the immunoregulatory actions of IFN-gamma versus IFN-alpha/beta on functions of different T cell populations. Cell Immunol. 1990 7;128(2):603–618. doi: 10.1016/0008-8749(90)90052-S 2162739 10.1016/0008-8749(90)90052-s

[35] Balmasova IP, Yushchuk ND, Mynbaev OA, Alla NR, Malova ES, Shi Z, et al Immunopathogenesis of chronic hepatitis B. World J Gastroenterol. 2014 10 21;20(39):14156–14171. doi: 10.3748/wjg.v20.i39.14156 25339804 10.3748/wjg.v20.i39.14156 PMC4202346

[36] Yates A, Callard R, Stark J. Combining cytokine signaling with T-bet and GATA-3 regulation in Th1 and Th2 differentiation: A model for cellular decision-making. J Theo Biol. 2004;231(2):181–196. doi: 10.1016/j.jtbi.2004.06.013 10.1016/j.jtbi.2004.06.013 15380383

[37] Weng WL, Liu Y, Chen JL, Huang T, Xu LJ, Godoy P, et al The Etiology of Liver Damage Imparts Cytokines Transforming Growth Factor *β*1 or Interleukin-13 as Driving Forces in Fibrogenesis. Hepathol. 2009;50(1):230–243. doi: 10.1002/hep.22934 10.1002/hep.22934 19441105

[38] Kim Y, Lawler S, Nowicki MO, Chiocca EA, Friedman A. A mathematical model for pattern formation of glioma cells outside the tumor spheroid core. J Theor Biol. 2009;260(3):359–371. doi: 10.1016/j.jtbi.2009.06.025 19596356 10.1016/j.jtbi.2009.06.025

[39] Terrault NA, Bzowej NH, Chang K, Hwang JP, Jonas MM, Murad MH. AASLD Guidelines for Treatment of Chronic Hepatitis B. Hepatology: Practice Guideline. 2015;00(00):1–23. 10.1002/hep.28156 PMC5987259 26566064

[40] Maloy KJ, Powrie F. Intestinal homeostasis and its breakdown in inflammatory bowel disease. Nature. 2011;474(7351):298–306. doi: 10.1038/nature10208 21677746 10.1038/nature10208

[41] Baumgart DC, Carding SR. Inflammatory bowel disease: cause and immunoliology. Lancet. 2007;369(9573):1627–1640. doi: 10.1016/S0140-6736(07)60750-8 17499605 10.1016/S0140-6736(07)60750-8

[42] Albeiroti S, Ayasoufi K, Hill DR, Shen B, de la Motte CA. Platelet hyaluronidase-2: An enzyme that translocates to the surface upon activation to function in extracellular matrix degradation. Blood. 2015;125:1460–1469. doi: 10.1182/blood-2014-07-590513 25411425 10.1182/blood-2014-07-590513 PMC4342357

[43] Baranova A, Lal P, Birerdinc A, Younossi ZM. Non-invasive markers for hepatic fibrosis. BMC Gastroenterol. 2011;11:91 doi: 10.1186/1471-230X-11-91 21849046 10.1186/1471-230X-11-91 PMC3176189

[44] Greco RM, Iocono JA, Ehrlich HP. Hyaluronic acid stimulates human fibroblast proliferation within a collagen matrix. J Cell Physiol. 1998;177:465–473. doi: 10.1002/(SICI)1097-4652(199812)177:3%3C465::AID-JCP9%3E3.0.CO;2-5 9808154 10.1002/(SICI)1097-4652(199812)177:3<465::AID-JCP9>3.0.CO;2-5

[45] Bai Q, An J, Wu X, You H, Ma H, Liu T, et al HBV promotes the proliferation of hepatic cells via the PDGF-B/PDGFR-*β* signaling pathway in vitro. Int J Mol Med. 2012 12;30(6):1443–50. doi: 10.3892/ijmm.2012.1148 23042547 10.3892/ijmm.2012.1148

[46] Fan JM, Ng YY, Hill PA, Nikolic-Paterson DJ, Mu W, Atkins RC, et al Transforming growth factor-beta regulates tubular epithelial-myofibroblast transdifferentiation in vitro. Kidney Int. 1999;56:1455–1467. doi: 10.1046/j.1523-1755.1999.00656.x 10504497 10.1046/j.1523-1755.1999.00656.x

[47] Thomas NJ, Watts KL, Akram KM, Forsyth NR, Spiteri MA. Idiopathic pulmonary fibrosis: immunohistochemical analysis provides fresh insights into lung tissue remodelling with implications for novel prognostic markers. Int J Clin Exp Pathol. 2012;5:58–71. 22295148 PMC3267487

[48] Friedman SL. Hepatic stellate cells: Pretean, multifunctional, and enigmatic cells of the liver. Physiol Rev. 2008;88:125–172. doi: 10.1152/physrev.00013.2007 18195085 10.1152/physrev.00013.2007 PMC2888531

[49] Lu P, Takai K, Weaver VM, Werb Z. Extracellualr matrix degradation and remodeling in development and disease. Cold Spring Harb Perspect Biol. 2011;3:1–25. doi: 10.1101/cshperspect.a005058 10.1101/cshperspect.a005058 PMC3225943 21917992

[50] Murray LA, Chen Q, Kramer MS, Hesson DP, Argentieri RL, et al TGF-beta driven lung fibrosis in macrophage dependent and blocked by Serum amyloid P. Int J Biochem Cell Biol. 2011;43:154–162. doi: 10.1016/j.biocel.2010.10.013 21044893 10.1016/j.biocel.2010.10.013

[51] Hao W, Komar HM, Hart PA, Conwell DL, Lesinski GB, Friedman A. Mathematical model of chronic pancreatitis. PNAS. 2017 3 30;114(19):5011–5016. doi: 10.1073/pnas.1620264114 28439020 10.1073/pnas.1620264114 PMC5441692

[52] Hao W, Rovin BH, Friedman A. Mathematical model of renal interstitial fibrosis. Proc Natl Acad Sci. 2014;111(39):14193–14198. doi: 10.1073/pnas.1413970111 25225370 10.1073/pnas.1413970111 PMC4191765

[53] Zan Y, Zhang Y, Tien P. Hepatitis B virus e antigen induces activation of rat hepatic stellate cells. Biochem Biophys Res Commun. 2013 6;435(3):391–6. doi: 10.1016/j.bbrc.2013.04.098 23665329 10.1016/j.bbrc.2013.04.098

[54] Wada T, Yokoyama H, Matsushima K, Kobayashi K. Monocyte chemoattractant protein-1: does it play a role in diabetic nephropathy? Nephrol Dial Transplant. 2003;18:457–459. doi: 10.1093/ndt/18.3.457 12584260 10.1093/ndt/18.3.457

[55] Hong M, Chou Y, Wu Y, Tsai K, Hu C, Jeng K, et al Transforming growth factor-*β*1 suppresses hepatitis B virus replication by the reduction of hepatocyte nuclear factor-4*α* expression. PLoS ONE. 2012 1;71(1):1–13. 10.1371/journal.pone.0030360 PMC3262823 22276183

[56] Ciupe SM, Ribiero RM, Nelson PW, Perelson AS. Modeling the mechanisms of acute hepatitis B virus infection. J Theor Biol. 2007;247:23–35. doi: 10.1016/j.jtbi.2007.02.017 17428501 10.1016/j.jtbi.2007.02.017 PMC1994818

[57] Colombatto P, Civitano L, Bizzarri R, Oliveri F, Choudhury S, Gieschke R, et al A multiphase model of the dynamics of HBV infection in HBeAg-negative patients during pegylated interferon-*α*2a, lamivudine and combination therapy. Anti-viral Therapy. 2006;11:197–212. 16640101

[58] Lewina S, Ribeiro R, Walters T, Lau G, Bowden S, Locarnini S, et al Analysis of hepatitis B viral load decline under potent therapy: complex decay profiles observed. Hepatology. 2001;34:1012–1020. doi: 10.1053/jhep.2001.28509 11679973 10.1053/jhep.2001.28509

[59] Lau G, Tsiang M, Hou J, Yuen S, Carman W, Zhang L, et al Combination therapy with lamivudine and famciclovir for chronic hepatitis B infected Chinese patients: a viral dynamics study. Hepatology. 2000;32:394–399. doi: 10.1053/jhep.2000.9143 10915748 10.1053/jhep.2000.9143

[60] Ciupe SM, Catlla AJ, Forde J, Schaeffer DG. Dynamics of hepatitis B virus infection: What causes viral clearance? Mathematical Population Studies. 2011;18:87–105. doi: 10.1080/08898480.2011.564563

[61] Ciupe SM, Ribiero RM, Perelson AS. Antibody Responses during Hepatitis B Viral Infection. PLoS Comp Biol. 2014;10(7):1–16. doi: 10.1371/journal.pcbi.1003730 10.1371/journal.pcbi.1003730 PMC4117427 25078553

[62] Kamyad AV, Akbari R, heydari AA, Heydari A. Mathematical Modeling of Transmission Dynamics and Optimal Control of Vaccination and Treatment for Hepatitis B Virus. Comp Math Methods Med. 2014 475451;2014:1–15. doi: 10.1155/2014/475451 10.1155/2014/475451 PMC4000643 24812572

[63] Zhao S, Su Z, Lu Y. A mathematical model of hepatitis B virus transmission and its application for vaccination strategy in china. Int J Epidemiol. 2000 8;29(4):744–752. doi: 10.1093/ije/29.4.744 10922354 10.1093/ije/29.4.744

[64] Friedman A, Hao W. Mathematical Modeling of Liver Fibrosis. Mat Biosc Eng. 2017 2;14(1):143–164. doi: 10.3934/mbe.2017010 10.3934/mbe.2017010 27879125

[65] Siewe N, Yakubu AA, Satoskar AR, Friedman A. Immune Response to Infection by Leishmania: A Mathematical Model. Mathematical Biosciences. 2016 3;276:28–43. doi: 10.1016/j.mbs.2016.02.015 26987853 10.1016/j.mbs.2016.02.015

[66] Siewe N, Yakubu AA, Satoskar AR, Friedman A. Granuloma Formation in Leishmaniasis: A Mathematical Model. J Theor Biol. 2017 1;412:48–60. doi: 10.1016/j.jtbi.2016.10.004 27769685 10.1016/j.jtbi.2016.10.004

[67] Biermer M, Puro R, Schneider RJ. Tumor Necrosis Factor Alpha Inhibition of Hepatitis B Virus Replication Involves Disruption of Capsid Integrity through Activation of NF-*κ*B. J Virol. 2003 4;77(7):4033–4042. doi: 10.1128/JVI.77.7.4033-4042.2003 12634363 10.1128/JVI.77.7.4033-4042.2003 PMC150632

[68] Veremeyko T, Siddigui S, Sotnikov I, Yung A, Ponomarev ED. IL-4/IL-13-dependent and independent expression of miR-124 and its contribution to M2 phenotype of monocytic cells in normal conditions and during allergic inflammation. PLoS One. 2013 12;8(12):1–13. doi: 10.1371/journal.pone.0081774 10.1371/journal.pone.0081774 PMC3864800 24358127

[69] Geissmann CADFMGGEOJSKSSACGLF. Monitoring of blood vessels and tissues by a population of monocytes with patrolling behavior. Science. 2007 Aout;3(317):666–670. 10.1126/science.1142883 17673663

[70] Italiani P, Boraschi D. From Monocytes to M1/M2 Macrophages: Phenotypical vs. Functional Differentiation. Front Immunol. 2014 10;5(514):1–22. 25368618 10.3389/fimmu.2014.00514 PMC4201108

[71] Yang J, Zhang L, Yu C, Yang X, Wang H. Monocyte and macrophage differentiation: circulation inflammatory monocyte as biomarker for inflammatory diseases. Biomark Res. 2014 1;2(1):1–9. doi: 10.1186/2050-7771-2-1 24398220 10.1186/2050-7771-2-1 PMC3892095

[72] Chen HY, Chen ZX, Huang RF, Lin N, Wang XZ. Hepatitis B virus X protein activates human hepatic stellate cells through upregulating TGF*β*1. Genet Mol Res. 2014 10;13(4):8645–56. doi: 10.4238/2014.October.27.4 25366754 10.4238/2014.October.27.4

[73] Deepak P, Kumar S, Kishore D, Acharya A. IL-13 from Th2-type cells supresses induction of antigen-specific Th1 immunity in a T-cell lymphoma. Int Immunol. 2010;22(1):53–63. doi: 10.1093/intimm/dxp114 19951958 10.1093/intimm/dxp114

[74] Camelo A, Dunmore R, Sleeman MA, Clarke DL. The epithelium in idiopathic pulmonary fibrosis: breaking the barrier. Front Pharmacol. 2014;4(173). doi: 10.3389/fphar.2013.00173 24454287 10.3389/fphar.2013.00173 PMC3887273

[75] Clarke DL, Carruthers AM, Mustelin T, Murray LA. Matrix regulation of idiopathic pulmonary fibrosis: the role of enzymes. Fibrogenesis Tissue Repair. 2013;6(20). doi: 10.1186/1755-1536-6-20 24279676 10.1186/1755-1536-6-20 PMC4176485

[76] Janssen HLA, Berk L, Schalm SW, Heijtink RA, Hess G, Rossol S, et al Antiviral effect of prolonged intermittent lymphobastoid alpha interferon treatment in chronic hepatitis B. Gut. 1992;33:1094–1098. doi: 10.1136/gut.33.8.1094 1398234 10.1136/gut.33.8.1094 PMC1379449

[77] d’Onofrio A, Gandolfi A. The response to antiangiogenic anticancer drugs that inhibit endothelial cell proliferation. Appl Math Comput. 2006 10;181(2):1155–1162.

[78] d’Onofrio A, Gandolfi A, Rocca A. The dynamics of tumour-vasculature interaction suggests low-dose, time-dense anti-angiogenic schedulings. Cell Prolif. 2009 6;42(3):317–329. doi: 10.1111/j.1365-2184.2009.00595.x 19438898 10.1111/j.1365-2184.2009.00595.x PMC6760821

[79] d’Onofrio A, Gandolfi A. Chemotherapy of vascularised tumours: role of vessel density and the effect of vascular “pruning”. J Theor Biol. 2010 5;264(2):253–265. doi: 10.1016/j.jtbi.2010.01.023 20100496 10.1016/j.jtbi.2010.01.023

[80] WebMD. Drugs and Medications: HEPSERA. https://wwwwebmdcom/drugs/2/drug-64166/hepsera-oral/details 2005–2018;.

[81] Drug com: Know more Be Sure. Adefovir. https://wwwdrugscom/ppa/adefovirhtml 2000–2018;.

[82] WebMD. Interferons for Chronic Hepatitis B. https://wwwwebmdcom/hepatitis/interferons-for-chronic-hepatitis-b 2014;.

[83] Paul N, Han S. Combination Therapy for Chronic Hepatitis B: Current Indications. Curr Hepatitis Rep. 2011;10:98–105. doi: 10.1007/s11901-011-0095-1 10.1007/s11901-011-0095-1 PMC3085106 21654909

[84] Crump KS, Hoel DG, Langley CH, Peto R. Fundamental carcinogenic processes and their implications for low dose risk assessment. Cancer Res. 1976;36(9 pt1):2973–1979. 975067

[85] Altshuler B. Modeling of dose-response relationships. Env Health Perspec. 1981;42:23–27. doi: 10.1289/ehp.814223 10.1289/ehp.814223 PMC1568781 7333256

[86] Vanderberg LN, Colborn T, Hayes TB, Heindel JJ, Jacobs DR Jr, Lee DH, et al Hormones and endocrine-disrupting chemicals: low-dose effects nonmonotonic dose responses. Endocrine Rev. 2012;33(3):378–455. doi: 10.1210/er.2011-1050 22419778 10.1210/er.2011-1050 PMC3365860

[87] Lai X, Friedman A. Combination therapy of cancer with cancer vaccine and immune checkpoint inhibitors: A mathematical model. PLoS ONE. 2017 5;12(5):1–24. doi: 10.1371/journal.pone.0178479 10.1371/journal.pone.0178479 PMC5444846 28542574

[88] Dusheiko G. Side effects of alpha interferon in chronic hepatitis C. Hepatology. 1997 9;26(3 Suppl 1):112S–121S. doi: 10.1002/hep.510260720 9305675 10.1002/hep.510260720

[89] Huang R, Hao Y, Zhang J, Wu C. Interferon-alpha plus adefovir combination therapy versus interferon-alpha monotherapy for chronic hepatitis B treatment: A meta-analysis. Hepatol Res. 2013;43:1040–1051. 23356962 10.1111/hepr.12058

