# Supplementary material for: Chronic hepatitis B virus and liver fibrosis: A mathematical model
Source: PLoS One. 2018 Apr 10;13(4):e0195037. doi: 10.1371/journal.pone.0195037 (PMC5892900; doi:10.1371/journal.pone.0195037)
Supplement: S1 File — Parameter estimates (Section A in S1 File), parameter values (Tables C–F in S1 File) and numerical methods used (Section B in S1 File). (PDF) [file pone.0195037.s001.pdf]

Supporting Information – Chronic Hepatitis B Virus and Liver Fibrosis: A Mathematical Model

Avner Friedman[1] and Nourridine Siewe[2,*],

1 Mathematical Biosciences Institute & Department of Mathematics, The Ohio State University, Columbus, Ohio, United States of America
2 National Institute for Mathematical and Biological Synthesis, University of Tennessee, Knoxville, Tennessee, United States of America

* nourridine@aims.ac.za

A Parameter Estimates

Estimates of K_X and $\tilde{K}_X$

In an expression of the form $Y\dfrac{X}{X+K_X}$ where Y is activated by X, the half-saturation parameter K_X is taken to be the approximate steady state concentration of species X. Hence in steady state equations this factor is equal to 1/2. When species X does not reach a steady state, the parameter K_X will be taken approximately as the average concentration of X over the computational time; we refer to K_X as the "time-average" state of X.

In an expression of the form $Y\dfrac{1}{1+X/\tilde{K}_X}$ where Y is inhibited by X, the parameter $\tilde{K}_X$ is typically of the same order of magnitude as K_X. For simplicity we shall take $\tilde{K}_X = K_X$.

Estimates for the diffusion coefficients D_X

Diffusion coefficients of polymerase. Young [1] established the following formula for estimating the diffusion coefficients of a protein p:

$$D_p = \frac{M_V^{1/3}}{M_p^{1/3}} D_V, \qquad (1)$$

where M_V and D_V are respectively the molecular weight and diffusion coefficient of VEGF, M_p is the molecular weight of p, $M_V = 24$kDa [2] and $D_V = 8.64 \times 10^{-2}$ cm^2 d^{-1} [3]. The following table lists the molecular weights of the proteins in our model [2], and their corresponding diffusion coefficients computed from Formula (40).

X :	A	C_3	G	$I_{1\beta}$	I_2	I_4	I_6	I_{10}
MW (kDa):	0.273	10	27.3	30.7	17.6	17.5	23.7	20.5
D_X (10^{-2} cm^2 d^{-1}):	38.42	11.57	8.28	7.96	9.58	9.6	8.68	9.11

X :	I_{12}	I_{13}	I_α	I_γ	T_α	T_β	M_P	T_P
MW (kDa):	37.2	15.8	21.7	19.3	25.6	22.3	54	24
D_X (10^{-2} cm^2 d^{-1}):	7.47	9.93	8.94	9.29	8.46	8.85	6.59	8.64

MW = Molecular Weight.

Diffusion coefficients of cells and extracellular virus. According to [4–6], the diffusion coefficient of macrophages is $D_M = 8.64 \times 10^{-7}$ cm^2 d^{-1}. For simplicity, we assume that all cell types have the same diffusion coefficient, so that

$$D_X = 8.64 \times 10^{-7} \text{ cm}^2 \text{ d}^{-1},$$

for $X = M_1,\ M_{1i},\ M_2,\ M_{2i},\ H,\ H_i,\ T_1,\ T_2,\ f,\ m.$

The diffusion coefficient of extracellular *Mycobacterium tuberculosis* (Mtb) is approximately 10^{-6} cm^2 d^{-1} [7]. Since the diameter of a Mtb is significantly larger than the diameter of HBV virus, we take the diffusion coefficient of extracellular HBV virus to be larger than that of extracellular Mtb,

$$D_{V_e} = 1.2 \times 10^{-5} \text{ cm}^2 \text{ d}^{-1}.$$

Estimates for time-average states of cells

We assume that the cells in the human liver constitute 80% of the total liver mass, as in mice [8], so that their average density is 0.8 g/cm^3.

'Time-average' state of HSCs (H and H_i). HSCs constitute about 7% of the total liver cell population [9]. Hence

$$H + H_i = 7\% \text{ of } 0.8 = 0.056 \text{ g/cm}^3.$$

Since HSCs are pro-inflammatory, we assume that the density of healthy HSCs in fibrotic time-average state is larger than that of infected HSCs and take $H = 3H_i$. Hence in fibrotic time-average state

$$H = 0.042 \text{ g/cm}^3 \qquad \text{and} \qquad H_i = 0.014 \text{ g/cm}^3.$$

'Time-dependent' steady states of macrophages ($M_1,\ M_{1i},\ M_2$ and M_{2i}). Hepatic macrophages constitute 10–15% of total liver cell population [10]. Accordingly we take the density of macrophages to be

$$M_1 + M_{1i} + M_2 + M_{2i} = 0.1 \text{ g/cm}^3.$$

Now, since HBV promotes M2–like activation in macrophages in cell culture studies [11], we take $M_2 + M_{2i} = 4(M_1 + M_{1i})$ so that

$$M_1 + M_{1i} = 0.02 \text{ g/cm}^3 \qquad \text{and} \qquad M_2 + M_{2i} = 0.08 \text{ g/cm}^3$$

in time-average state. Since M1 macrophages are pro-inflammatory we take $M = 3M_{1i}$, and, since M2 macrophages are anti-inflammatory, we take $M_{2i} = 3M_2$, so that, in time-average state,

$$M_1 = 0.015 \text{ g/cm}^3,\ M_{1i} = 0.005 \text{ g/cm}^3,\ M_2 = 0.02 \text{ g/cm}^3,\ M_{2i} = 0.06 \text{ g/cm}^3.$$

'Time-dependent' steady state of T cells (T_1 and T_2). Th1 cells are activated by contact with M1 macrophage and Th2 cells are activated by contact with M2 macrophages. Accordingly we take in time-average state

$$T_1 = 80\%(M_1 + M_{1i}) = 0.16 \text{ g/cm}^3, \qquad T_2 = 80\%(M_2 + M_{2i}) = 0.64 \text{ g/cm}^3.$$

'Time-dependent' steady state of ECM (ρ), fibroblast (f) and myofibroblast (m). We assume that the carrying capacity of ECM in homeostasis is $\rho_0 = 0.003$ g/cm^3 [12], while in chronic HBV, under fibrotic conditions, the time-average state of ρ is

$$\rho = 0.001 \text{ g/cm}^3.$$

In HBV, fibroblasts differentiate into contractile and secretory myofibroblasts that contribute to tissue repair during liver fibrosis [13]. Accordingly we take in time-average states,

$$m = 3f, \qquad f + m \text{ in } 0\text{–}0.024 \text{ g/cm}^3.$$

Thus

$$f \text{ in } 0\text{–}0.006 \text{ g/cm}^3 \text{ and } m \text{ in } 0\text{–}0.018 \text{ g/cm}^3.$$

Estimates for time-average states of cytokines

In what follows, we assume that the concentration of the cytokines in the tissue is larger, by a factor of up to 20, than the concentration of the cytokines in serum.

- K_G: The level of serum PDGF at different stages of HBV–liver fibrosis ranges between 4.2–4.8$\times 10^{-12}$ g/cm^3 [14]. We accordingly take G in fibrotic time-average state in the tissue to be $G = K_G = 2.5 \times 10^{-11}$ g/cm^3.
- K_{H_A}: The level of serum HA in patients with chronic HBV ranges between 2-4$\times 10^{-7}$ g/cm^3 [15]. We take the H_A fibrotic time-average state to be $H_A = K_{H_A} = 2 \times 10^{-6}$ g/cm^3.
- $K_{1\beta}$: The levels of serum IL-1 at clinical diagnosis in controlled (C) and non-controlled (NC) cases of HBV $86 \pm 26 \times 10^{-12}$ g/cm^3 [16]. We take $I_{1\beta} = K_{1\beta} = 5.6 \times 10^{-10}$ g/cm^3 in fibrotic time-average state.
- K_2: The level of serum IL-2 in patients with chronic HBV ranges between 1–5$\times 10^{-8}$ g/cm^3 [17]. We take $I_2 = K_2 = 2.5 \times 10^{-7}$ g/cm^3 in fibrotic time-average state.
- K_4: The level of serum IL-4 at different stages in patients with chronic HBV infection ranges between 1–6$\times 10^{-11}$ g/cm^3 [18]. We take I_4 in fibrotic time-average state to be $I_4 = K_4 = 3 \times 10^{-10}$ g/cm^3.
- K_6: The level of serum IL-6 at different stages in patients with chronic HBV infection ranges between 1.4–16.1$\times 10^{-12}$ g/cm^3 [19]. We take the I_6 fibrotic time-average state to be $I_6 = K_6 = 8 \times 10^{-11}$ g/cm^3.
- K_{10}: The level of serum IL-10 at different stages in patients with chronic HBV infection ranges between 1.75–5$\times 10^{-10}$ g/cm^3 [18]. We take the I_{10} fibrotic time-average state to be $I_{10} = K_{10} = 2.5 \times 10^{-9}$ g/cm^3.
- K_{12}: The level of serum IL-12 at different stages in patients with chronic HBV infection ranges between 1–7$\times 10^{-10}$ pg/cm^3 [20]. We take the I_{12} fibrotic time-average state to be $I_{12} = K_{12} = 3.5 \times 10^{-9}$ g/cm^3.
- K_{13}: The level of serum IL-13 in patients with chronic HCV infection ranges between 40–80 pg/cm^3 [21]. We assume that the level of IL-13 in patients with chronic HBV is similar to that of patient with chronic HCV. We take the I_{13} fibrotic time-average state to be $I_{13} = K_{13} = 4 \times 10^{-10}$ g/cm^3.
- K_α: The level of serum IFN-α/β in patients with chronic HCV infection ranges between 50–300 pg/cm^3 [22]. We assume that the level of IFN-α/β in patients with chronic HBV is similar to that of patient with chronic HCV. We take the I_α fibrotic time-average state to be $I_\alpha = K_\alpha = 1.5 \times 10^{-9}$ g/cm^3.
- K_γ: The level of serum IFN-γ at different stages in patients with chronic HBV infection ranges between 20–60 pg/cm^3 [18]. We take the I_γ fibrotic time-average state to be $I_\gamma = K_\gamma = 3 \times 10^{-10}$ g/cm^3.
- K_{T_α}: The level of serum TNF-α at different stages in patients with chronic HBV infection ranges between 1.5–47 pg/cm^3 [19]. We take the T_α fibrotic time-average state to be $T_\alpha = K_{T_\alpha} = 2.35 \times 10^{-10}$ g/cm^3.
- K_{T_β}: The level of TGF-β1 in patients with chronic HCV ranges between 60–140 ng/cm^3 [17]. We assume that the level of TGF-β1 in patients with chronic HBV is similar to that of patient with chronic HCV. We take the T_β fibrotic time-average state to be $T_\beta = K_{T_\beta} = 7 \times 10^{-7}$ g/cm^3.

- K_{C_3}: The level of serum CCL3 at different stages in patients with chronic HBV infection ranges between 1–40 pg/cm^3 [23]. We take the C_3 fibrotic time-average state to be $C_3 = K_{C_3} = 2 \times 10^{-10}$ g/cm^3.

Estimates for activation rates of cells

Parameter Revision

In estimating some of the production rates, we use steady state equations with variables which are in time-average state. However, since the progression of HBV toward fibrosis involves a continuous transition from M1 to M2 macrophages, we shall need to accordingly revise the values of some of the parameters: A production rate of a cytokine produced by M2 macrophage should be increased, and a production rate of cytokine produced by M1 macrophage should be decreased.

Eqs. (1), (2), (29) and (32): We take the rate of polarization from M2 macrophage to M1 macrophage to be equal to that from M1 macrophage to M2 macrophage, $\lambda_{M_1M_2} = \lambda_{M_2M_1}$. We also assume that the rate of infection of M1 macrophage by HBV is equal to the rate of infection of HSC, since both cells are pro-inflammatory, but smaller than the rate of infection of M2 macrophage by HBV, since M2 is anti-inflammatory; we take $\lambda_{M_2M_{2i}} = 2\lambda_{M_1M_{1i}} = 2\lambda_{HH_i}$. Furthermore, we assume that the growth rate of HBV in M2i macrophage is larger than the growth rates of HBV in M1i macrophage and Hi HSC and take $\lambda_{V_iM_{2i}} = 2\lambda_{V_iM_{1i}} = 2\lambda_{V_iH_i}$. We shall use Eqs. (1), (2), (29) and (32) in order to estimate $\lambda_{M_1M_2}$, $\lambda_{M_1M_{1i}}$, $\lambda_{V_iM_{1i}}$ and the parameter N_V=(mass of 1 virus)/(mass of one cell). To do that we note that $M_{10} = 0.02$ g/cm^3 [7]. Recall that $M_1 = 0.015$ g/cm^3, $M_2 = 0.02$ g/cm^3, $M_{1i} = 0.005$ g/cm^3 in time-average state, and assume that the total density of intracellular and extracellular virus are 2×10^{-9} g/cm^3 and $V_e = 10^{-11}$ g/cm^3, respectively. Assuming that $V_{i1} = V_{i2} = V_{iH} = \frac{2}{3}10^{-9}$ g/cm^3, we can write the steady state of Eqs. (1), (2), (29) and (32) in the following form

$$
\begin{aligned}
\frac{M_{10} - M_1}{2} + \frac{2\lambda_{M_1M_2}}{3}(M_2 - M_1) - \lambda_{M_1M_{1i}} V_e M_1 - \mu_{M_1} M_1 = 0 \\
\lambda_{M_1M_{1i}} V_e M_1 - \mu_{M_1}(1 + \mu_{V_i1} V_{i1}) M_{1i} = 0 \\
\frac{\lambda_{V_iM_{1i}}}{16} M_{1i} + \lambda_{M_1M_{1i}} N_V M_1 V_e - \mu_{M_1}(1 + \mu_{V_i1} V_{i1}) N V_{1i} - \frac{\mu_{M_{1i}M}}{2} V_{i1} = 0 \\
N[\mu_{M_1}(1 + \mu_{V_i1} V_{i1}) V_{i1} + \mu_{M_2}(1 + \mu_{V_i1} V_{i2}) N V_{2i} + \mu_H (1 + \mu_{V_i1} V_{iH}) N V_{iH}] \\
-(\lambda_{M_1M_{1i}} M_1 + 2\lambda_{M_1M_{1i}} M_2 + \lambda_{M_1M_{1i}} H) N_V V_e - \mu_{V_e} V_e = 0,
\end{aligned}
$$

from which we compute that

$$\lambda_{M_1M_2} = 0.21\,\mathrm{d}^{-1},\ \lambda_{M_1M_{1i}} = 10^9\,\mathrm{d}^{-1},\ \lambda_{V_iM_{1i}} = 4.8{\times}10^{-5}\,\mathrm{d}^{-1},\ N_V = 10^{-6}\,\mathrm{cm}^3/\mathrm{g}.$$

It then also follows that $\lambda_{M_2M_1} = 0.21$ d^{-1}, $\lambda_{HH_i} = 10^9$ d^{-1}, $\lambda_{M_2M_{2i}} = 2 \times 10^9$ d^{-1}, $\lambda_{V_iH_i} = 4.8 \times 10^{-5}$ d^{-1}, $\lambda_{V_iM_{2i}} = 9.6 \times 10^{-5}$ d^{-1}.

Eq (5): Assuming that $\lambda_{HG} = \lambda_{HT_\beta}$, we have in steady state $A_H + \lambda_{HG} H - \frac{\lambda_{HH_i}}{2} H - \mu_H H = 0$, where $\mu_H = 0.0166$ d^{-1} [24], $A_H = 0.002$ g/cm^3, $H = 0.042$ g/cm^3 and $\lambda_{HH_i} = 10^9$ d^{-1}. Hence $\lambda_{HG} = \lambda_{HT_\beta} = 0.069$ d^{-1}.

Eq. (7) and (8): We assume that the rate of activation of Th2 cells is larger than that of Th1 cells, and take $\lambda_{T_2M_2} = 4\lambda_{T_1M_1} = 4\lambda_{T_1I_2}$. In steady state,

$\frac{\lambda_{T_1M_1}}{16}T_0 + \frac{\lambda_{T_1M_1}}{4}T_1 - \mu_{T_1}T_1 = 0$ and $\frac{\lambda_{T_1M_1}}{2}T_0 - \mu_{T_2}T_2$, where
$\mu_{T_1} = 0.197$ d^{-1}, $\mu_{T_2} = 0.197$ d^{-1} [6],
$T_0 = 0.001$ g/cm^3 [7], $T_1 = 0.016$ g/cm^3, $T_2 = 0.064$ g/cm^3 in time-average state.
Hence $\lambda_{T_1M_1} = \lambda_{T_1I_2} = 0.191$ d^{-1}, $\lambda_{T_2M_2} = 0.504$ d^{-1}.

The values of several production rates were obtained under steady state assumptions. However the HBV is a progressive disease and we expect the transition from M1 macrophage to M2 macrophage to increase towards fibrosis. Accordingly, by the section on Parameter Revision above, we increase the values of $\lambda_{M_1M_2}$, $\lambda_{T_1M_1}$ and $\lambda_{T_2M_2}$, so that $\lambda_{M_1M_2} = 5.425$ d^{-1}, $\lambda_{T_1M_1} = 38.105$ d^{-1} and $\lambda_{T_2M_2} = 286$ d^{-1}. We also decrease the values of $\lambda_{M_2M_1}$, λ_{HG} and λ_{HT_β}, so that $\lambda_{M_2M_1} = 2.17$ d^{-1}, $\lambda_{HG} = 2.45 \times 10^{-2}$ d^{-1}, $\lambda_{HT_\beta} = 2.45 \times 10^{-2}$ d^{-1}.

Eqs. (9) and (10): Baum *et al.* [25] reported that cardiac myofibroblasts could persist in the injured heart for over 20 weeks post injury as the scar matures. We assume that liver myofibroblasts have a larger lifespan than cardiac myofibroblasts and take the lifespan of the liver myofibroblasts to be 300 days. Hence

$$\mu_m = 1/300 = 3.33 \times 10^{-3} \text{ per day.}$$

Assuming that $\lambda_{mfT_\beta} = \lambda_{mfG}$, Eq. (10) in steady state leads to $\lambda_{mfT_\beta} = 3\mu_m$. In steady state, Eq (9) becomes $\lambda_{fH_A} = 2\mu_f + \lambda_{mfT_\beta} + \lambda_{mfG}$, where $\mu_f = 0.0166$ d^{-1} [8,26]. Hence $\lambda_{mfT_\beta} = \lambda_{mfG} = 0.01$ d^{-1} and $\lambda_{fH_A} = 5.32 \times 10^{-2}$ d^{-1}.

Estimates for proliferation rates of cytokines

We estimate the various parameters using their corresponding steady state equations. The values are then adjusted to reflect the respective levels of cytokines during the course of chronic HBV (as given by the K_X's above).

Eq. (13): PDGF enhances the formation of myofibroblast and hence it increases ECM in HBV infection. We therefore assume that PDGF is produced by infected M_2 macrophages at a larger rate than by healthy M_2 macrophages, and take $\lambda_{GM_{2i}} = 2\lambda_{GM_2}$. In fibrotic time-average state, $\lambda_{GM_2} = \frac{\mu_G K_G}{M_2 + 2M_{2i}}$, where $\mu_G = 4.16$ d^{-1} [6], $M_2 = 0.02$ g/cm^3 and $M_{2i} = 0.06$ g/cm^3. Hence $\lambda_{GM_2} = 7.43 \times 10^{-9}$ d^{-1} and $\lambda_{GM_{2i}} = 1.49 \times 10^{-9}$ d^{-1}.

Eq. (14): Since HA enhances the proliferation of fibroblast which, in turn, produces fibroblast, we assume that infected HSCs produce HA at a larger rate than healthy HSCs, and take $\lambda_{H_AH_i} = 2\lambda_{H_AH}$. In fibrotic time-average state, $\lambda_{H_AH} = \frac{\mu_{H_A}K_{H_A}}{H + 2H_i}$, where $\mu_{H_A} = 0.1$ d^{-1} [24], $H = 0.042$ g/cm^3 and $H_i = 0.014$ g/cm^3. Hence $\lambda_{H_AH} = 2.86 \times 10^{-6}$ d^{-1} and $\lambda_{H_AH_i} = 5.71 \times 10^{-6}$ d^{-1}.

Eq. (15): Since IL-1β inhibits the immune response of T cells by blocking the production of IFN-α, we assume that M2i macrophages produce IL-1β at a larger rate than M2 macrophages, and take $\lambda_{I_{1\beta}M_{2i}} = 2\lambda_{I_{1\beta}M_2}$. In fibrotic time-average state, $\lambda_{I_{1\beta}M_2} = \frac{4\mu_{I_{1\beta}}I_{1\beta}}{M_2 + 2M_{2i}}$, where $\mu_{I_{1\beta}} = 6.65$ d^{-1} [6], $M_2 = 0.02$ g/cm^3 and $M_{2i} = 0.06$ g/cm^3. Hence $\lambda_{I_{1\beta}M_2} = 1.06 \times 10^{-7}$ d^{-1} and $\lambda_{I_{1\beta}M_{2i}} = 2.12 \times 10^{-7}$ d^{-1}. By the section on Parameter Revision above, we increase the values of the steady state parameters $\lambda_{I_{1\beta}M_2}$ and $\lambda_{I_{1\beta}M_{2i}}$ and take $\lambda_{I_{1\beta}M_2} = 4.77 \times 10^{-7}$ d^{-1} and $\lambda_{I_{1\beta}M_{2i}} = 9.6 \times 10^{-7}$ d^{-1}.

Eq. (16): In time-average state in health, $\lambda_{I_2T_1} = \frac{\mu_{I_2} I_2}{T_1}$, where $\mu_{I_2} = 2.376$ d^{-1} [6] and $T_1 = 0.016$ g/cm^3. Hence $\lambda_{I_2T_1} = 3.71 \times 10^{-5}$ d^{-1}.

Eq. (17): We assume that IL-4 is secreted by M2i macrophages at a larger rate than by healthy macrophages, and take $\lambda_{I_4M_{2i}} = 2\lambda_{I_4M_2}$. We also assume that the secretion of IL-4 by Th2 is at a lower rate than the secretion by M2 macrophages, and take $\lambda_{I_4T_2} = \lambda_{I_4M_2}/2$. In time-average state, $\lambda_{I_4T_2} = \frac{\mu_{I_4} I_4}{2M_2 + 4M_{2i} + T_2}$, where $\mu_{I_4} = 50$ d^{-1} [6,27], $M_2 = 0.02$ g/cm^3, $M_{2i} = 0.06$ g/cm^3 and $T_2 = 0.064$ g/cm^3. Hence $\lambda_{I_4M_2} = 8.8 \times 10^{-9}$ d^{-1}, $\lambda_{I_4M_{2i}} = 1.76 \times 10^{-8}$ d^{-1} and $\lambda_{I_4T_2} = 4.36 \times 10^{-9}$ d^{-1}.

Eq. (18): Since IL-6 inhibits the replication of intracellular viruses, we assume that it is secreted by M1i macrophages at a larger rate than by M1 macrophages, and take $\lambda_{I_6M_{1i}} = 2\lambda_{I_6M_1}$. In time-average state, $\lambda_{I_6M_{1i}} = \frac{2\mu_{I_6} I_6}{3(M_1 + 2M_{1i})}$, where $\mu_{I_6} = 0.173$ d^{-1} [6,28], $M_1 = 0.015$ g/cm^3 and $M_{1i} = 0.005$ g/cm^3. Hence $\lambda_{I_6M_{1i}} = 7.38 \times 10^{-10}$ d^{-1} and $\lambda_{I_6M_1} = 3.69 \times 10^{-10}$ d^{-1}. By the section on Parameter Revision above, we decrease the value of the parameter $\lambda_{I_6M_{1i}}$, taking $\lambda_{I_6M_{1i}} = 4.92 \times 10^{-10}$ d^{-1}.

Eq. (19): We assume that IL-10 is secreted by M2i macrophages at a larger rate than by M2 macrophages, and take $\lambda_{I_{10}M_{2i}} = 2\lambda_{I_{10}M_2}$. We also assume that the secretion of IL-10 by Th2 is at a lower rate than by healthy M2 macrophages and take $\lambda_{I_{10}T_2} = \lambda_{I_{10}M_2}/2$. In fibrotic time-average state, $\lambda_{I_{10}T_2} = \frac{\mu_{I_{10}} I_{10}}{2M_2 + 4M_{2i} + T_2}$, where $\mu_{I_{10}} = 8.32$ d^{-1} [6,29], $M_2 = 0.02$ g/cm^3, $M_{2i} = 0.06$ g/cm^3 and $T_2 = 0.064$ g/cm^3. Hence $\lambda_{I_{10}M_2} = 1.21 \times 10^{-7}$ d^{-1}, $\lambda_{I_{10}M_{2i}} = 2.42 \times 10^{-7}$ d^{-1} and $\lambda_{I_{10}T_2} = 6.05 \times 10^{-8}$ d^{-1}.

Eq. (20): Since IL-12 activates the T cells immune response, we assume that it is secreted by M1i pro-inflammatory macrophages at a larger rate than by M1 macrophages. We take $\lambda_{I_{12}M_{1i}} = 2\lambda_{I_{12}M_1}$. In time-average state, $\lambda_{I_{12}M_{1i}} = \frac{4\mu_{I_{12}} I_{12}}{M_1 + 2M_{1i}}$, where $\mu_{I_{12}} = 1.38$ d^{-1} [6,30], $M_1 = 0.015$ g/cm^3 and $M_{1i} = 0.005$ g/cm^3. Hence $\lambda_{I_{12}M_1} = 7.73 \times 10^{-7}$ d^{-1} and $\lambda_{I_{12}M_{1i}} = 1.54 \times 10^{-6}$ d^{-1}. By the section on Parameter Revision above, we decrease the values of the parameters $\lambda_{I_{12}M_1}$ and $\lambda_{I_{12}M_{1i}}$. Hence $\lambda_{I_{12}M_1} = 5.15 \times 10^{-6}$ d^{-1}, $\lambda_{I_{12}M_{1i}} = 1.28 \times 10^{-6}$ d^{-1}.

Eq. (21): As in the case of IL-10, we assume that IL-13 is secreted by M2i macrophages at a larger rate than by M2 macrophages, and take $\lambda_{I_{13}M_{2i}} = 2\lambda_{I_{13}M_2}$. We also assume that the secretion of IL-13 by Th2 is at a lower rate than M2 macrophages and take $\lambda_{I_{13}T_2} = \lambda_{I_{13}M_2}/2$. In time-average state, $\lambda_{I_{13}T_2} = \frac{\mu_{I_{13}} I_{13}}{2M_2 + 4M_{2i} + T_2}$, where $\mu_{I_{13}} = 12.47$ d^{-1} [6], $M_2 = 0.02$ g/cm^3, $M_{2i} = 0.06$ g/cm^3 and $T_2 = 0.064$ g/cm^3 in average steady state. Hence $\lambda_{I_{13}M_2} = 2.8 \times 10^{-8}$ d^{-1}, $\lambda_{I_{13}M_{2i}} = 5.6 \times 10^{-8}$ d^{-1} and $\lambda_{I_{13}T_2} = 1.4 \times 10^{-8}$ d^{-1}.

Eq. (22): In steady state in health, we assume that Th1 and Th2 cells secrete IFN-α at equal rates, so that, with $\lambda_{I_\alpha T_1} = \lambda_{I_\alpha T_2}$, $\lambda_{I_\alpha T_1} = \frac{\mu_{I_\alpha} I_\alpha}{T_1 + T_2}$, where $\mu_{I_\alpha} = 1.66$ d^{-1} [7], $T_1 = 0.016$ g/cm^3 and $T_2 = 0.064$ g/cm^3 in average steady state. Hence $\lambda_{I_\alpha T_1} = \lambda_{I_\alpha T_2} = 6.2 \times 10^{-8}$ d^{-1}.

Eq. (23): In time-average state in health, $\lambda_{I_\gamma T_1} = \dfrac{\mu_{I_\gamma} I_\gamma}{T_1}$, where $\mu_{I_\gamma} = 2.16$ d^{-1} [31] and $T_1 = 0.016$ g/cm^3. Hence $\lambda_{I_\gamma T_1} = 8.1 \times 10^{-8}$ d^{-1}.

Eq. (24): TNF-α inhibits the replication of intracellular viruses, and we assume that it is secreted by M1 and M1i macrophages at a larger rate than by M2i macrophages. We take $\lambda_{T_\alpha M_{1i}} = 2\lambda_{T_\alpha M_1}$, and $\lambda_{T_\alpha M_{2i}} = \lambda_{T_\alpha M_1}/2$. In fibrotic time-average state, $\lambda_{T_\alpha M_{1i}} = \dfrac{4\mu_{T_\alpha} T_\alpha}{M_1 + 2M_{1i} + 2M_{2i}}$, where $\mu_{T_\alpha} = 199$ d^{-1} [32], $M_1 = 0.015$ g/cm^3, $M_{1i} = 0.005$ g/cm^3 and $M_{2i} = 0.06$ g/cm^3. Hence $\lambda_{T_\alpha M_1} = 1.29 \times 10^{-6}$ d^{-1}, $\lambda_{T_\alpha M_{1i}} = \lambda_{T_\alpha M_{2i}} = 2.58 \times 10^{-6}$ d^{-1}. By the section of Parameter Revision above, we decrease the steady state value of the parameter $\lambda_{T_\alpha M_1}$, taking $\lambda_{T_\alpha M_1} = 2.58 \times 10^{-7}$ d^{-1}.

Eq. (25): Since TGF-β inhibits the replication of intracellular viruses, we assume that the secretion of TGF-β by M2i macrophages has a larger rate than that by M2 macrophages, taking $\lambda_{T_\beta M_{2i}} = 2\lambda_{T_\beta M_2}$. In time-average state, $\lambda_{T_\beta M_2} = \dfrac{\mu_{T_\beta} T_\beta}{2(M_2 + 2M_{2i})}$, where $\mu_{T_\beta} = 333$ d^{-1} [6], $\lambda_{T_\beta I_{13}} = 2$, $M_2 = 0.02$ g/cm^3 and $M_{2i} = 0.06$ g/cm^3. Hence $\lambda_{T_\beta M_2} = 8.325 \times 10^{-4}$ d^{-1} and $\lambda_{T_\beta M_{2i}} = 1.67 \times 10^{-3}$ d^{-1}. By the section on Parameter Revision, we increase the steady state value of the parameter $\lambda_{T_\beta M_{2i}}$, taking $\lambda_{T_\beta M_{2i}} = 2.51 \times 10^{-3}$ d^{-1}.

Eq. (28): CCL3 enhances the differentiation of monocytes into healthy macrophages [33, 34], and we assume that all infected cells secrete CCL3 at equal rates, $\lambda_{C_3 M_{1i}} = \lambda_{C_3 H_i} = \lambda_{C_3 M_{2i}}$. Then, in time-average state, $\lambda_{C_3 M_{2i}} = \dfrac{\mu_{C_3} C_3 + 0.5 d_{C_3 M_1} M_1}{H_i + M_{1i} + M_{2i}}$, where $\mu_{C_3} = 1.73$ d^{-1} [6], $d_{C_3 M_1} = 2.08 \times 10^{-4}$ d^{-1}, $M_1 = 0.015$ g/cm^3 and $M_{1i} = 0.005$ g/cm^3, $M_{2i} = 0.06$ g/cm^3 and $H_i = 0.014$ g/cm^3 in time-average state. Hence $\lambda_{C_3 M_{1i}} = \lambda_{C_3 M_{2i}} = \lambda_{C_3 H_i} = 4.38 \times 10^{-9}$ d^{-1}.

B Numerical Methods

We are using the scheme introduced in [24]. Accordingly, in order to illustrate our numerical method, we consider the following diffusion equation:

$$\frac{\partial X}{\partial t} - D_X \nabla^2 X = F_X \text{ in } \Omega,$$

where the right-hand side accounts for all the 'active' terms. Let X_{ij}^n denote a numerical approximation of $X(ih_x, jh_y, n\tau)$, where h_x and h_y are the stepsize in the x and y directions respectively, and τ is the time stepsize. Then a discretization is derived by the explicit Euler five-point difference scheme, i.e.,

$$\frac{X_{ij}^{n+1} - X_{ij}^n}{\tau} - D_X \left(\frac{X_{i+1,j}^n + X_{i-1,j}^n - 2X_{ij}^n}{h_x^2} + \frac{X_{i,j+1}^n + X_{i,j-1}^n - 2X_{ij}^n}{h_y^2} \right) = F_X(X_{ij}^n) \text{ in } \Omega.$$

In order to make the scheme stable, we take $\tau \le \dfrac{h^2}{4D_X}$, namely $\tau = 0.1\dfrac{h^2}{D_X}$, where $h = h_x = h_y$.

Parameters	Descriptions	Values	References
D_M	diffusion coefficient of macrophages	8.64×10^{-7} cm^2 d^{-1}	[4–6]
D_H	diffusion coefficient of HSCs	8.64×10^{-7} cm^2 d^{-1}	est.
D_T	diffusion coefficient of T cells	8.64×10^{-7} cm^2 d^{-1}	[4–6]
D_f	diffusion coefficient of f	1.47×10^{-6} cm^2 d^{-1}	[6]
D_m	diffusion coefficient of m	1.47×10^{-5} cm^2 d^{-1}	[6]
D_G	diffusion coefficient of G	8.28×10^{-2} cm^2 d^{-1}	est.
D_{H_A}	diffusion coefficient of H_A	0.1 d^{-1}	[24]
$D_{I_{1\beta}}$	diffusion coefficient of $I_{1\beta}$	7.96×10^{-2} cm^2 d^{-1}	est.
D_{I_2}	diffusion coefficient of I_2	9.58×10^{-2} cm^2 d^{-1}	est.
D_{I_4}	diffusion coefficient of I_4	9.6×10^{-2} cm^2 d^{-1}	est.
D_{I_6}	diffusion coefficient of I_6	8.68×10^{-2} cm^2 d^{-1}	est.
$D_{I_{10}}$	diffusion coefficient of I_{10}	9.11×10^{-2} cm^2 d^{-1}	est.
$D_{I_{12}}$	diffusion coefficient of I_{12}	7.47×10^{-2} cm^2 d^{-1}	est.
$D_{I_{13}}$	diffusion coefficient of I_{13}	9.93×10^{-2} cm^2 d^{-1}	est.
D_{I_α}	diffusion coefficient of I_α	8.94×10^{-2} cm^2 d^{-1}	est.
D_{I_γ}	diffusion coefficient of I_γ	9.29×10^{-2} cm^2 d^{-1}	est.
D_{T_α}	diffusion coefficient of T_α	8.46×10^{-2} cm^2 d^{-1}	est.
D_{T_β}	diffusion coefficient of T_β	8.85×10^{-2} cm^2 d^{-1}	est.
D_{M_P}	diffusion coefficient of M_P	6.59×10^{-2} cm^2 d^{-1}	est.
D_{T_P}	diffusion coefficient of T_P	8.64×10^{-2} cm^2 d^{-1}	est.
D_{C_3}	diffusion coefficient of C_3	11.53×10^{-2} cm^2 d^{-1}	est.
D_{V_e}	diffusion coefficient of V_e	1.2×10^{-5} cm^2 d^{-1}	est.

Table C Diffusion coefficients.

Parameters	Descriptions	Values	References
μ_{M_1}	death rate of M_1	0.02 d^{-1}	[6,31]
μ_{M_2}	death rate of M_2	0.008 d^{-1}	[6,31]
μ_H	death rate of H	1.66×10^{-2} d^{-1}	[24]
μ_{T_1}	death rate of T_1	1.97×10^{-1} d^{-1}	[6,8,35]
μ_{T_2}	death rate of T_2	1.97×10^{-1} d^{-1}	[6,8,35]
μ_f	death rate of f	1.66×10^{-2} d^{-1}	[8,26]
μ_m	death rate of m	3.33×10^{-3} d^{-1}	est.
μ_ρ	death rate of ρ	0.37 d^{-1}	[6]
$\mu_{M_{1i}M}$	killing rate of V_i1 by macrophages	788 d^{-1}	est.
$\mu_{M_{2i}M}$	killing rate of V_i2 by macrophages	788 d^{-1}	est.
μ_{H_iM}	killing rate of V_iH by macrophages	788 d^{-1}	est.
μ_{V_e}	death rate of V_e	474.4 d^{-1}	[36]
$\mu_{V_{i1}}$	death rate of V_i in M_1	1.4×10^9 cm^3/g	est.
$\mu_{V_{i2}}$	death rate of V_i in M_2	2.8×10^9 cm^3/g	est.
$\mu_{V_{iH}}$	death rate of V_i in H	1.4×10^9 cm^3/g	est.
μ_G	degradation rate of G	4.16 d^{-1}	[6]
μ_{H_A}	degradation rate of H_A	0.1 d^{-1}	[24]
$\mu_{I_{1\beta}}$	degradation rate of I_β	6.65 d^{-1}	[6]
μ_{I_2}	degradation rate of I_2	2.376 d^{-1}	[6]
μ_{I_4}	degradation rate of I_4	50 d^{-1}	[6,27]
μ_{I_6}	degradation rate of I_6	0.173 d^{-1}	[6,28]
$\mu_{I_{10}}$	degradation rate of I_{10}	8.32 d^{-1}	[6,29]
$\mu_{I_{12}}$	degradation rate of I_{12}	1.38 d^{-1}	[6,30]
$\mu_{I_{13}}$	degradation rate of I_{13}	12.47 d^{-1}	[6]
μ_{I_α}	degradation rate of I_α	1.66 d^{-1}	[7]
μ_{I_γ}	degradation rate of I_γ	2.16 d^{-1}	[31]
μ_{T_α}	degradation rate of T_α	199 d^{-1}	[32]
μ_{T_β}	degradation rate of T_β	333 d^{-1}	[6]
μ_{M_P}	degradation rate of M_P	4.32 d^{-1}	[6]
μ_{T_P}	degradation rate of T_P	21.6 d^{-1}	[6]
μ_{C_3}	degradation rate of C_3	1.73 d^{-1}	[6]
μ_A	degradation rate of A	2.23 d^{-1}	[37,38]

Table D. Death rates, killing rates and degradation rates of variables.

Parameters	Descriptions	Values	References
$\lambda_{M_2M_1}$	maximal rate of $M_2 \to M_1$ transition	0.21 d^{-1}	est.
$\lambda_{M_1M_2}$	maximal rate of $M_1 \to M_2$ transition	0.21 d^{-1}	est.
$\lambda_{M_1M_{1i}}$	infection rate of M_1	10^9 d^{-1}	est.
$\lambda_{M_2M_{2i}}$	infection rate of M_2	2×10^9 d^{-1}	est.
λ_{HH_i}	infection rate of H	10^9 d^{-1}	est.
$\lambda_{V_iM_{1i}}$	growth rate of V_i in M_{1i}	4.8×10^{-5} d^{-1}	est.
$\lambda_{V_iM_{2i}}$	growth rate of V_i in M_{2i}	4.8×10^{-5} d^{-1}	est.
$\lambda_{V_iH_i}$	growth rate of V_i in H_i	4.8×10^{-5} d^{-1}	est.
λ_{MI_4}	production rate of M_1 by I_4	2 d^{-1}	[7]
λ_{MI_γ}	production rate of M_1 by I_γ	2 d^{-1}	[7]
λ_{MT_α}	production rate of M_1 by T_α	2 d^{-1}	[7]
$\lambda_{MI_{13}}$	production rate of M_1 by I_{13}	2 d^{-1}	[7]
λ_{HG}	production rate of H by G	6.9×10^{-2} d^{-1}	est.
λ_{HT_β}	production rate of H by T_β	6.9×10^{-2} d^{-1}	est.
$\lambda_{T_1M_1}$	production of rate of T_1 by M_1 and I_{12}	0.191 d^{-1}	est.
$\lambda_{T_1I_2}$	production rate of T_1 by I_2	0.191 d^{-1}	est.
$\lambda_{T_2M_2}$	production rate of T_2	0.504 d^{-1}	est.
λ_{fH_A}	production rate of f by H_A	0.133 d^{-1}	[24]
λ_{mfT_β}	activation rate of f due to T_β	0.01 d^{-1}	[39]
λ_{mfG}	activation rate of f due to G	0.01 d^{-1}	[39]
$\lambda_{\rho f}$	activation rate of ρ due to f	3×10^{-3} d^{-1}	[8, 40]
$\lambda_{\rho m}$	activation rate of ρ due to m	0.576 d^{-1}	[8, 40]
$\lambda_{\rho H}$	activation rate of ρ by healthy HSCs	6.6×10^{-3} d^{-1}	[8, 40]
$\lambda_{\rho H_i}$	activation rate of ρ by infected HSCs	0.66 d^{-1}	[8, 40]
$\lambda_{\rho T_\beta}$	fraction for activation rate of ρ due to T_β	95	[8]
λ_{GM_2}	production of PDGF by M_2	7.43×10^{-10} d^{-1}	est.
$\lambda_{GM_{2i}}$	production of PDGF by M_{2i}	1.49×10^{-9} d^{-1}	est.
λ_{H_AH}	production rate of H_A by H	2.86×10^{-6} d^{-1}	est.
$\lambda_{H_AH_i}$	production rate of H_A by H_i	5.72×10^{-6} d^{-1}	est.
$\lambda_{I_{1\beta}M_2}$	production rate of $I_{1\beta}$ by M_2	1.064×10^{-7} d^{-1}	est.
$\lambda_{I_{1\beta}M_{2i}}$	production rate of $I_{1\beta}$ by M_{2i}	2.13×10^{-7} d^{-1}	est.
$\lambda_{I_2T_1}$	production rate of I_2 by T_1	3.71×10^{-5} d^{-1}	est.
$\lambda_{I_4T_2}$	production rate of I_4 by T_2	4.36×10^{-9} d^{-1}	est.
$\lambda_{I_4M_2}$	production rate of I_4 by M_2	8.72×10^{-9} d^{-1}	est.
$\lambda_{I_4M_{2i}}$	production rate of I_4 by M_2	1.74×10^{-8} d^{-1}	est.
$\lambda_{I_6M_1}$	production rate of I_6 by M_{1i}	3.69×10^{-10} d^{-1}	est.
$\lambda_{I_6M_{1i}}$	production rate of I_6 by M_{1i}	7.38×10^{-10} d^{-1}	est.
$\lambda_{I_{10}M_2}$	production of I_{10} by M_2	1.21×10^{-7} d^{-1}	est.
$\lambda_{I_{10}M_{2i}}$	production of I_{10} by M_{2i}	2.42×10^{-7} d^{-1}	est.
$\lambda_{I_{10}T_2}$	production rate of I_{10} by T_2	6.07×10^{-8} d^{-1}	est.
$\lambda_{I_{12}M_1}$	production rate of I_{12} by M_1	7.73×10^{-7} d^{-1}	est.
$\lambda_{I_{12}M_{1i}}$	production rate of I_{12} by M_{1i}	1.54×10^{-6} d^{-1}	est.
$\lambda_{I_{13}M_2}$	production rate of I_{13} by M_2	2.9×10^{-8} d^{-1}	est.
$\lambda_{I_{13}M_{2i}}$	production rate of I_{13} by M_2	5.8×10^{-8} d^{-1}	est.
$\lambda_{I_{13}T_2}$	production rate of I_{13} by T_2	1.45×10^{-8} d^{-1}	est.
$\lambda_{I_\gamma T_1}$	production rate of I_γ by T_1	8.1×10^{-8} d^{-1}	est.
$\lambda_{I_\alpha T_1}$	production rate of I_α by T_1	6.23×10^{-8} d^{-1}	est.
$\lambda_{I_\alpha T_2}$	production rate of I_α by T_2	6.23×10^{-8} d^{-1}	est.
$\lambda_{T_\alpha M_1}$	production rate of T_α by M_1	1.29×10^{-6} d^{-1}	est.
$\lambda_{T_\alpha M_{1i}}$	production rate of T_α by M_{1i}	2.58×10^{-6} d^{-1}	est.
$\lambda_{T_\alpha M_{2i}}$	production rate of T_α by M_{2i}	2.58×10^{-6} d^{-1}	est.
$\lambda_{T_\beta M_2}$	production rate of T_β by M_2	8.33×10^{-4} d^{-1}	est.
$\lambda_{T_\beta M_{2i}}$	production rate of T_β by M_{2i}	1.67×10^{-3} d^{-1}	est.
$\lambda_{T_\beta I_{13}}$	fraction for induction of production rate of T_β by I_{13}	2	[24, 35]
$\lambda_{M_PM_2}$	production rate of MMP by M_2	3×10^{-4} d^{-1}	[8, 24]
$\lambda_{M_PM_{2i}}$	production rate of MMP by M_{2i}	9×10^{-3} d^{-1}	[8, 24]
$\lambda_{T_PM_2}$	production rate of TIMP by M_2	6×10^{-5} d^{-1}	[8, 24]
$\lambda_{T_PM_{2i}}$	production rate of TIMP by M_{2i}	5.4×10^{-4} d^{-1}	[8, 24]
$\lambda_{C_3H_i}$	production rate of CCL3 by H_i	4.38×10^{-9} d^{-1}	est.
$\lambda_{C_3M_{1i}}$	production rate of CCL3 by M_{1i}	4.38×10^{-9} d^{-1}	est.
$\lambda_{C_3M_{2i}}$	production rate of CCL3 by M_{2i}	4.38×10^{-9} d^{-1}	est.
λ_S	growth fraction of scar	1	[24]
λ_{SM_P}	fraction of effect of MMP on growth of scar	1	[24]

Table E. Activation rates, growth rates and production rates of variables.

Parameters	Descriptions	Values	Units
K_{C_3}	C_3 value for 1/2 maximum effect of C_3	2×10^{-10} g/cm^3	est.
K_G	G value for 1/2 maximum effect of G	2.5×10^{-11} g/cm^3	est.
K_{H_A}	H_A value for 1/2 maximum effect of H_A	2×10^{-6} g/cm^3	[24]
K_{M_1}	M_1 value for 1/2 maximum effect of M_1	0.015 g/cm^3	est.
K_{M_2}	M_2 value for 1/2 maximum effect of M_2	0.02 g/cm^3	est.
K_{M_P}	M_P value for 1/2 maximum effect of M_P	10^{-6} g/cm^3	[24]
K_{T_1}	T_1 value for 1/2 maximum effect of T_1	0.016 g/cm^3	est.
K_{T_2}	T_2 value for 1/2 maximum effect of T_2	0.064 g/cm^3	est.
K_{T_β}	T_β value for 1/2 maximum effect of T_β	7×10^{-7} g/cm^3	[8]
K_{T_α}	T_α value for 1/2 maximum effect of T_α	2.35×10^{-10} g/cm^3	[6]
$K_{1\beta}$	$I_{1\beta}$ value for 1/2 maximum effect of $I_{1\beta}$	5.6×10^{-10} g/cm^3	[7]
K_2	I_2 value for 1/2 maximum effect of I_2	2.5×10^{-7} g/cm^3	[6]
K_4	I_4 value for 1/2 maximum effect of I_4	3×10^{-10} g/cm^3	[6, 24]
K_6	I_6 value for 1/2 maximum effect of I_6	8×10^{-11} g/cm^3	[6, 24]
K_{10}	I_{10} value for 1/2 maximum effect of I_{10}	2.5×10^{-9} g/cm^3	[6]
K_{12}	I_{12} value for 1/2 maximum effect of I_{12}	3.5×10^{-9} g/cm^3	[6]
K_{13}	I_{13} value for 1/2 maximum effect of I_{13}	4×10^{-10} g/cm^3	[6]
K_α	I_α value for 1/2 maximum effect of I_α	1.5×10^{-9} g/cm^3	[6]
K_γ	I_γ value for 1/2 maximum effect of I_γ	3×10^{-10} g/cm^3	[6]
K_{V_e}	V_e value for 1/2 maximum effect of V_e	5×10^{-8} g/cm^3	[31, 41]
$\tilde{K}_{T_\beta}$	T_β value for 1/2 inhibitory effect of T_β	7×10^{-7} g/cm^3	est.
$\tilde{K}_{T_\alpha}$	T_α value for 1/2 inhibitory effect of T_α	2.35×10^{-10} g/cm^3	est.
$\tilde{K}_{1\beta}$	$I_{1\beta}$ value for 1/2 inhibitory effect of $I_{1\beta}$	5.6×10^{-10} g/cm^3	[7]
$\tilde{K}_{10}$	I_{10} value for 1/2 inhibitory effect of I_{10}	2.5×10^{-9} g/cm^3	est.
$\tilde{K}_{13}$	I_{13} value for 1/2 inhibitory effect of I_{13}	4×10^{-10} g/cm^3	est.
$\tilde{K}_\alpha$	I_α value for 1/2 inhibitory effect of I_α	1.5×10^{-9} g/cm^3	est.
$\tilde{K}_\gamma$	I_γ value for 1/2 inhibitory effect of I_γ	3×10^{-10} g/cm^3	est.
A_H	recruitment rate of H	0.002 g/cm3 d^{-1}	[24]
N	number of viruses that are released when a cell dies	10	est.
N_V	proportion of viruses that are internalized by a cell	10^{-6} cm^3/g	est.
χ_{C_3}	chemotaxis rate	10 g/cm^3	[31]
$d_{C_3M_1}$	internalization rate of C_3 by M1 macrophage	1.16×10^{-4} d^{-1}	[8]
$d_{\rho M_P}$	degradation rate of ECM due to MMP	2.59×10^7 cm^{3}g^{-1}d^{-1}	[8, 42]
$d_{M_PT_P}$	binding rate of MMP to TIMP	4.98×10^5 cm^{3}g^{-1}d^{-1}	[5]
$d_{T_PM_P}$	binding rate of TIMP to MMP	1.04×10^6 cm^{3}g^{-1}d^{-1}	[5]
δ	small parameter for numerical purposes	2×10^{-5}	[31]
ρ^*	ECM density in health	3.26×10^{-3} g/cm^3	[35]
ρ_0	ECM saturation	0.003 g/cm^3	[8]
M_{10}	source of M1 macrophages	0.02 g/cm^3	[7]
M_{20}	source of M1 macrophages	0.02 g/cm^3	[7]
T_0	source of naive T cells	0.001 g/cm^3	[7]

Table F. Values of other parameters.

Acknowledgement

This research was supported by the Mathematical Biosciences Institute of The Ohio State University. This work was conducted while Nourridine Siewe was a Postdoctoral Fellow at the National Institute for Mathematical and Biological Synthesis, an Institute sponsored by the National Science Foundation through NSF Award #DBI-1300426, with additional support from The University of Tennessee, Knoxville.

References

1. Young ME. Estimation of diffusion coefficients of proteins. Biotech Bioeng. 1980;XXII:947–955.

2. Hornbeck PV, Zhang B, Murray B, Kornhauser JM, Latham V, Skrzypek E. PhosphoSitePlus, 2014: mutations, PTMs and recalibrations. Nucleic Acids Research – PhosphoSitePlus(R) (PSP, http://wwwphosphositeorg/). 2015;43:D512–D520.

3. Liao KL, Bai XF, Friedman A. Mathematical modeling of interleukin-27 induction of anti-tumor T cells response. PLoS ONE. 2014;9(3).

4. Friedman A, Hao W. A mathematical model of atherosclerosis with reverse cholesterol transport and associated risk factors. Bull Math Biol. 2015;77(5):758–781.

5. Hao W, Friedman A. The LDL-HDL Profile Determines the Risk of Atherosclerosis: A Mathematical Model. PLoS ONE. 2014;9(3):60–65.

6. Hao W, Crouser ED, Friedman A. Mathematical Model of Sarcoidosis. PNAS. 2014 November;111(45):16065–16070.

7. Hao W, Schlesinger LS, Friedman A. Modeling Granuloma in Response to Infection in the Lung. PLoS ONE. 2016;11(3):1–26.

8. Hao W, Rovin BH, Friedman A. Mathematical model of renal interstitial fibrosis. Proc Natl Acad Sci. 2014;111(39):14193–14198.

9. Blouin A, Bolender RP, Weibel ER. Distribution of organelles and membranes between hepatocytes and nonhepatocytes in the rat liver parenchyma. A stereological study. J Cell Biol. 1977;72:441–455.

10. Li H, You H, Fan X, Jia J. Hepatic macrophages in liver fibrosis: pathogenesis and potential therapeutic targets. BMJ Open Gastroenterol. 2016;3(1):1–4.

11. Bility MT, Chiang L, Zhang Z, Luan Y, Li F, Chi L, et al. Hepatitis B Virus Infection and Immunopathogenesis in a Humanized Mouse Model: Induction of Human-Specific Liver Fibrosis and M2–Like Macrophages. PLOS Pathogens. 2014 March;10(3):1–14.

12. Miller CC, Godeau G, Lebreton-DeCoster C, Desmoulière A, Pellat B, Dubertret L, et al. Validation of a morphometric method for evaluating fibroblast numbers in normal and pathologic tissues. Exp Dermatol. 2003;12(4):403–11.

13. Hinz B, Phan SH, Thannickal VJ, Galli A, Bochaton-Piallat M, Gabbiani G. The Myofibroblast: One Function, Multiple Origins. Am J Pathol. 2007;170(6):1807–1816.

14. Zhou J, Deng Y, Yan L, Zhao H, Wang G. Serum platelet-derived growth factor BB levels: a potential biomarker for the assessment of liver fibrosis in patients with chronic hepatitis B. Int J Infec Dis. 2016;49:94–99.

15. Weng HL, Wang BE, Jia JD, Wu WF, Xian JZ, Mertens PR, et al. Effect of Interferon-Gamma on Hepatic Fibrosis in Chronic Hepatitis B Virus Infection: A Randomized Controlled Study. Clin Gastroentorol Hepatol. 2005;3:819–828.

16. Halfon P, Bourlière M, Pénaranda G, Deydier R, Renou C, Botta-Fridlund D, et al. Accuracy of hyaluronic acid level for predicting liver fibrosis stages in patients with hepatitis C virus. Compar Hepatol. 2005;4(6):1–7.

17. Tang JT, Fang JY, Gu WQ, Li EL. T cell immune response is correlated with fibrosis and inflammatory activity in hepatitis B cirrhotics. World J Gastroentor. 2006;12(19):3015–3019.

18. Wang Y, Yu W, Shen C, Wang W, Zhang L, Liu F, et al. Predictive Value of Serum IFN-γ inducible Protein-10 and IFN-γ/IL-4 Ratio for Liver Fibrosis Progression in CHB Patients. Scientific Reports. 2017;7(40404):1–11.

19. Mourtzikou A, Alepaki M, Stamouli M, Pouliakis A, Skliris A, Karakitsos P. Evaluation of serum levels of IL-6, TNF-γ, IL-10, IL-2 and IL-4 in patients with chronic hepatitis. Immunologia. 2014;33(2):41–50.

20. He D, Yan G, Wang Y. Serum levels of interleukin-12 in various clinical states with hepatitis B virus infection. Cell Immunol. 2012;272:162–165.

21. Weng WL, Liu Y, Chen JL, Huang T, Xu LJ, Godoy P, et al. The Etiology of Liver Damage Imparts Cytokines Transforming Growth Factor β1 or Interleukin-13 as Driving Forces in Fibrogenesis. Hepathol. 2009;50(1):230–243.

22. Yatsuhashi H, Fujino T, Matsumoto T, Inoue O, Koga M, Yano M. Immunohistochemical analysis of hepatic interferon alpha-beta receptor level: relationship between receptor expression and response to interferon therapy in patients with chronic hepatitis C. J Hepathol. 1999;30:995–1003.

23. Heiberg IL, Winther TN, Paludan SR, Hogh B. Pattern recognition receptor responses in children with chronic hepatitis B virus infection. J Clin Virol. 2012;54:229–234.

24. Friedman A, Hao W. Mathematical Modeling of Liver Fibrosis. Mat Biosc Eng. 2017 February;14(1):143–164.

25. Baum J, Duffy HS. Fibroblasts and Myofibroblasts: What are we talking about? J Cardiovasc Pharmacol. 2011 April;57(4):376–379.

26. Tietz NW. Clinical Guide to Laboratory Tests. 3rd ed. Philadelphia; 1999.

27. Conlon PJ, Tyler S, Grabstein KH, Morrissey P. Interleukin-4 (B-cell stmulatory factor-1) augments the in vivo generation of cytotoxic cells in immunosuppressed animals. Biotechnol Ther. 1989;1(1):31–41.

28. Lu ZY, Brailly H, Wijdenes J, Bataille R, Rossi JF. Measurement of whole body interleukin-6 (IL-6) production: prediction of the efficacy of anti-IL-6 treatments. Blood. 1995;86(8):3123–3131.

29. Li L, Elliott JF, Mosmann TR. IL-10 inhibits cytokine production, vascular leakage, and swelling during T helper 1 cell-induced delayed-type hypersensitivity. J Immunol. 1994;153(9):3967–3978.

30. Bajetta E, Vechio MD, Mortarini R, Nadeau R, Rakhit A, Rimassa L, et al. Pilot Study of Subcutaneous Recombinant Human Interleukin 12 in Metastatic Melanoma. Clin Cancer Res. 1998 Jan;4:75–85.

31. Day J, Friedman A, Schlesinger LS. Modeling the immune rheostat of macrophages in the lung in response to infection. PNAS. 2009 June;p. 1–6.

32. Simo R, Barbosa-Desongles A, Lecube A, Hernandez C, Selva DM. Potential Role of Tumor Necrosis Factor-α in Downregulating Sex Hormone–Binding Globulin. Diabetes. 2012 February;61:372–382.

33. Vernon MA, Mylonas KJ, Hughes J. Macrophages and renal fibrosis. Semin Nephrol. 2010;30:302–317.

34. Wada T, Yokoyama H, Matsushima K, Kobayashi K. Monocyte chemoattractant protein-1: does it play a role in diabetic nephropathy? Nephrol Dial Transplant. 2003;18:457–459.

35. Hao W, Marsh C, Friedman A. A mathematical model of idiopathic pulmonary fibrosis. PLoS ONE. 2015;10(9).

36. Murray JM, Purcell RH, Wieland SF. The half-life of hepatitis B virions. Hepatol. 2006 November;44(5):1117–1121.

37. Chu CK. Recent Advances in Nucleosides: Chemistry and Chemotherapy. 1st ed. Chu CK, editor. Elsevier; 2002.

38. Baumgart DC, Carding SR. Inflammatory bowel disease: cause and immunoliology. Lancet. 2007;369(9573):1627–1640.

39. Wakefield LM, Winokur TS, Hollands RS, Christopherson K, Levinson AD, Sporn MB. Recombinant latent transforming growth factor beta 1 has a longer plasma half-life in rats than active transforming growth factor beta 1, and a different tissue distribution. J Clin Invest. 1990;86(6):1976–1984.

40. Mercapide J, Lopez De Cicco R, Castresana JS, Klein-Szanto AJ. Stromelysin-1/matrix metalloproteinase-3 (MMP-3) expression accounts for invasive properties of human astrocytoma cell lines. Int J Cancer. 2003;166(5):676–682.

41. Davis, Dulbecco, Eisen, Ginsberg. Bacterial Physiology: Microbiology. 2nd ed. Maryland: Harper and Row; 1973.

42. Kim Y, Lawler S, Nowicki MO, Chiocca EA, Friedman A. A mathematical model for pattern formation of glioma cells outside the tumor spheroid core. J Theor Biol. 2009;260(3):359–371.
